# Metagenomics Analysis to Investigate the Microbial Communities and Their Functional Profile During Cyanobacterial Blooms in Lake Varese

**DOI:** 10.1007/s00248-021-01914-5

**Published:** 2021-11-12

**Authors:** Isabella Sanseverino, Patrizia Pretto, Diana Conduto António, Armin Lahm, Chiara Facca, Robert Loos, Helle Skejo, Andrea Beghi, Franca Pandolfi, Pietro Genoni, Teresa Lettieri

**Affiliations:** 1 European Commission, Joint Research Centre (JRC), Via E. Fermi 2749, 21027 Ispra, VA Italy; 2 Biosearch Ambiente Srl, Via Tetti Gai 59, 10091 Alpignano, TO Italy; 3 Bioinformatics Project Support, P.za S.M. Liberatrice 18, 00153 Roma, Italy; 4 Department of Environmental Science, Informatics and Statistics, University Ca’ Foscari Venezia, Via Torino 155, 301702 Mestre, VE Italy; 5 ARPA, Agenzia Regionale Per La Protezione Dell’Ambiente Della Lombardia, Via Ippolito Rosellini 17, 20124 Milano, Italy

**Keywords:** Freshwater, Algal bloom, Microbial populations, Metagenomics, Water quality, Lyngbya

## Abstract

Toxic cyanobacterial blooms represent a natural phenomenon caused by a mass proliferation of photosynthetic prokaryotic microorganisms in water environments. Bloom events have been increasingly reported worldwide and their occurrence can pose serious threats to aquatic organisms and human health. In this study, we assessed the microbial composition, with a focus on Cyanobacteria, in Lake Varese, a eutrophic lake located in northern Italy. Water samples were collected and used for obtaining a 16S-based taxonomic profile and performing a shotgun sequencing analysis. The phyla found to exhibit the greatest relative abundance in the lake included Proteobacteria, Cyanobacteria, Actinobacteriota and Bacteroidota. In the epilimnion and at 2.5 × Secchi depth, Cyanobacteria were found to be more abundant compared to the low levels detected at greater depths. The blooms appear to be dominated mainly by the species *Lyngbya robusta*, and a specific functional profile was identified, suggesting that distinct metabolic processes characterized the bacterial population along the water column. Finally, analysis of the shotgun data also indicated the presence of a large and diverse phage population.

**Supplementary Information:**

The online version contains supplementary material available at 10.1007/s00248-021-01914-5.

## Introduction

Cyanobacteria are photosynthetic bacteria that are mostly found in freshwater systems. Due to their long evolutionary history, they have adapted to climatic, geochemical and anthropogenic changes. They have a key role in geochemical cycles together with picoplankton and other microorganisms belonging to the microbial loop and are also involved in maintaining the environmental balance and the biodiversity of microorganisms. The increasing anthropogenic eutrophication and climate changes are contributing to the intense proliferation of Cyanobacteria in waterbodies, resulting in bloom formations [1]. Global warming is expected to intensify this phenomenon; indeed, the predicted increase in air and water temperature and the frequent rainfall events alternated with longer periods of drought may favour the water stratification in waterbodies and the dominance of cyanobacterial blooms [2]. The dynamics of bloom events are not yet fully understood; however, it is generally accepted that external factors, such as water temperature, nutrient loading and light intensity, can influence the potential of their occurrence [1]. In addition to environmental elements, biotic interactions between Cyanobacteria and heterotrophic bacteria may also influence the bloom dynamics [3, 4]. These microbial interactions are still little explored but seem to play positive (e.g. nutrient exchange, including vitamins) or negative roles (e.g. cyanolytic properties) in the cyanobacterial proliferation [5–9]. For decades, scientific studies were mainly restricted to addressing the influence of physico-chemical parameters (e.g. nutrients, pH and temperature) on the blooms without focusing on variations in the composition of the microbial community during a bloom outbreak. Generally, Cyanobacteria grow faster at high temperature determining a cyanobacterial dominance in temperate waterbodies where the vertical stratification, one of the main physical parameters, is intensified, leading to the occurrence of algal bloom events [10–13]. Phosphorus (P) and nitrogen (N) are also considered among the main factors responsible for the cyanobacterial proliferation [14, 15]. Indeed, eutrophication can result in algal bloom formations with a consequent increase in water turbidity and odour problems caused by the decomposition of algae. During a bloom, Cyanobacteria can also produce harmful toxins that can render water unsafe, cause fish mortality and affect human health [1]. Considering the ecological, economical and human health negative impacts of cyanobacterial blooms, their monitoring is crucial for an effective lake management, wherefore several predictive models have been already developed for forecasting cyanobacterial blooms in waterbodies [16]. Advances in sequencing technologies applied to environmental samples have improved our knowledge on the taxonomic composition of cyanobacterial communities, providing information on their relative abundance and their functional profile [17]. To date, many studies have been performed on Cyanobacteria using 16S sequencing with the aim to investigate the microbial community associated with blooms and factors promoting these events [18–23].

In this study, we used a sequencing approach to characterize the microbial community composition, with a focus on Cyanobacteria, in the Lake Varese (Italy) during two bloom events which occurred in two consecutive years. Lake Varese is considered one of the first and most evident examples of eutrophication in Europe [24]. The eutrophication process, in this lake, accelerated in the 1950s and was mainly caused by urban development and fertilization practices in agriculture. Initially classified as hypertrophic lake, following remedial actions aimed at reducing the P loading, Lake Varese is now in eutrophic status with bloom events occurring every year during the summer and early autumn [24–26]. Phytoplankton studies in Lake Varese were carried out only occasionally and first analyses revealed summer blooms associated with many genera of Cyanobacteria such as *Oscillatoria*, *Anabaena*, *Aphanizomenon*, *Gomphosphaeria*, *Leptolyngbya* and *Microcystis* [27–29]. However, a metagenomic approach has been never used before for providing a more comprehensive understanding on the composition of the microbial community in this lake. Information on the vertical distribution of bacteria and their functional profiles along the water column during blooms in Lake Varese is still missing. During summer 1997, a toxic algal bloom reported in this lake was characterized by the presence of a filamentous cyanobacterium, *Planktothrix* spp. FP1 strain, found to be responsible for the production of potent neurotoxins causing the human syndrome paralytic shellfish poisoning (PSP) [30]. Here, we present the first assessment of the microbial community composition in Lake Varese during cyanobacterial bloom events that occurred in the years 2016 and 2017, using a metagenomic approach. We also looked at the functional profile associated to the bacterial populations detected at the different water depths.

## Materials and Methods

### Study Area

**Fig. 1:**
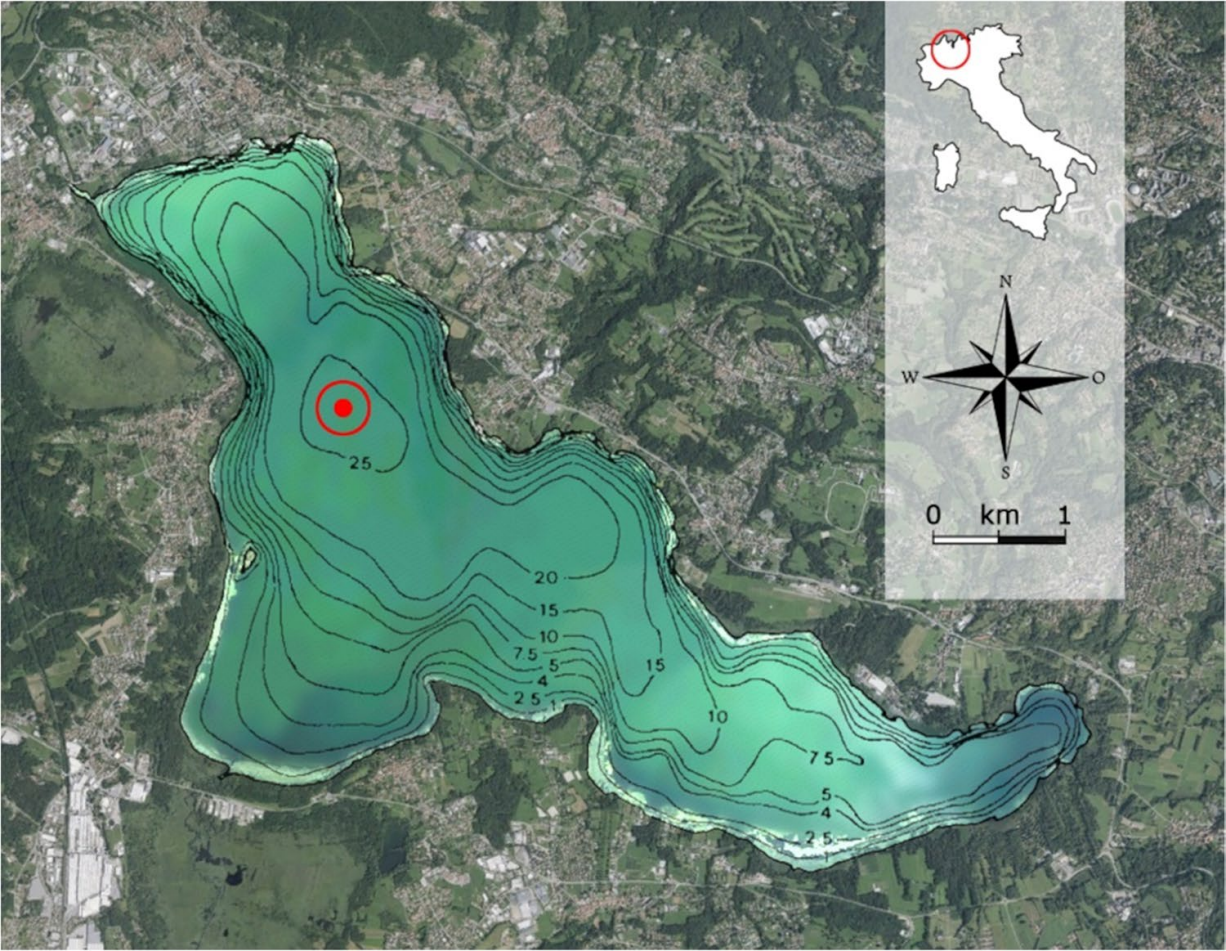
Location of the Lake Varese and lake bathymetry. The red dot in the figure indicates the deepest region of the lake where the samples were collected (45°49′.738N/008°43′.190E). The red circle in the upper map indicates the location where the Lake Varese is situated

Lake Varese (45°49′N; 8°44′E) is situated in northern Italy at the feet of the Alps mountain range at a mean altitude of 236 m above sea level (Fig. 1). It is a warm monomictic and eutrophic shallow lake, with a mean depth of 11 m, a maximum depth of 26 m, a surface area of 14.8 km^2^ and a theoretical renewal time of 1.7–1.9 years [25, 31].

### Sample Collection and Processing

Water collection was performed in Lake Varese at coordinates 45°49′0.738N/008°43′0.190E, corresponding to the deepest region of the lake with a maximum depth of 26 m, and indicated in Fig. 1 by a red dot. The collected samples were stored in thermic containers for the transport to laboratory facilities and immediately prepared for downstream analysis. To evaluate the dynamics of the microbial community in the lake, sampling campaigns were carried out mainly during the summer period and on a weekly basis, during two consecutive years (from 31 August to 5 October in 2016 and from 19 July to 4 October in 2017). Water samples were taken at 3 different water column depths: 0.5 m from surface (Epi depth, E), 13 m (Meso depth, M) and 2.5 times the Secchi disk depth (named in the paper as S). The euphotic depth was determined as 2.5 times the Secchi disk depth or the region where photosynthetically active radiation (PAR) was larger than 1% of the radiation determined immediately below the water surface. E samples represent the region with the highest oxygen and radiation exposure and M samples refer to the near anoxic region, where temperature and radiation are low. Secchi disk depth was determined using a Secchi disk. Part of the campaigns was performed by the department of the Regional Environmental Protection Agency of Lombardia (ARPA) which kindly provided us data and water samples for analysis.

Vertical profiles measuring physico-chemical parameters including pH, conductivity (CD), oxidation–reduction potential (ORP), PAR, dissolved oxygen (DO), oxygen saturation (OS) and water temperature (WT) were collected using a multi-parametric probe (Hydrolab DS5, Corr-Tek).

### Chlorophyll a, Cyanotoxins Analysis and Water Chemistry

A volume of 500 mL and 1 L of each water sample was filtered by GF/C membranes (Whatman) for chlorophyll *a* (Chl*a*) analysis. Filter membranes were folded, protected from light and stored at − 20 °C for a maximum of 1 day. For spectrophotometric analysis, 14 mL of methanol was added to each filter and samples were boiled at 70 °C for near 5 min (for the extraction of Chl*a*) and centrifuged for 7 min at 3500 rpm as described in [32], with slight modifications. The optical density (OD) was measured at 665 nm and 750 nm. Chl*a* content was determined by the following equation [32]:

$${Chl}a ({\mu g}/{L})=[ 13.9 * ({OD }665{ nm}-{ OD }750{ nm}) * v * d ] / P * V$$

where *v* is the added volume of methanol, *d* is the dilution factor (when applicable), *P* is the cuvette path in centimetres and *V* is the filtered water sample in litres.

Cyanotoxins concentrations (Microcystins, MCs; Saxitoxi, SX; and Anatoxin, AX) were quantified by enzyme-linked immunosorbent assay (ELISA) technique. Water samples were stored in glass vials at − 20 °C until analysis. Toxins quantification was performed using commercial kits (Abraxis, USA) according to supplier’s instructions.

Nutrients in the JRC laboratory were analysed with a Dionex ion chromatography system consisting of two pre-column-column-suppresser systems followed by electrochemical detection (NO_3_^−^ limit of detection_LOD = 0.049 mg/L, limit of quantification_LOQ = 0.086 mg/L; NH_4_^+^ LOD = 0.02 mg/L, LOQ = 0.02 mg/L; SO_4_^2−^ LOD = 0.002 mg/L, LOQ = 0.004 mg/L; total P_LOD = 0.05 mg/L) [33]. The analytical columns used were, for the cations, an IonPac CS15 with a guard column IonPac CG15 (column temperature of 45 °C) and, for the anions, an IonPac AS9 and a guard column IonPac AG9 (column temperature of 25 °C). The ion suppresser after the column increases the sensitivity by lowering the background noise. The solvent eluents (kept under Helium pressure — 6 bars — to eliminate oxygen) were methanesulfonic acid (20 mM) for the cations, and Na_2_CO_3_ (9 mM) for the anions, and a flow of 0.3 mL/min at approx. 1000 psi. Injection volume was 25 µL or 10 µL. In the BO laboratory, total P was measured according to the internal method MI-122 rev 8 2015. The method is based on the property of orthophosphate ions to react in an acidic environment with molybdate and antimonyl ions to form a phospho-antimonyl-molybdate reducible by ascorbic acid; the reduced heteropolyacid, containing hexa and tetravalent molybdenum, is soluble and shows a characteristic blue color: the final photometric determination is carried out at 800 nm. Organic phosphorus must be previously transformed into orthophosphate by hot oxidation with persulphate. The determination of the P concentration is performed through the use of the LCK 349 cuvette test (Hach Lange).

### DNA Extraction

For DNA extraction, aliquots of 250 mL or 500 mL of water were filtered through 0.22 µm nitrocellulose filter membranes (GSWP, Whatman). Filters were stored at − 20 °C (or − 80 °C for longer storage) until use. DNA extraction was performed according to the protocol described in Kisand et al. [34] except for the lyticase incubation. Briefly, each frozen filter was thawed, incubated in 5 mL of 50 mM KH_2_PO_4_ buffer (pH 7.5) and shaken overnight (160 rpm) at 4 °C. The following day, the KH_2_PO_4_ buffer was recovered and filters were transferred to a tube with 3 mL of fresh 50 mM KH_2_PO_4_ to be sonicated at 60 °C for a total of 15 min. Filters were discarded, and sonicated and not sonicated buffers were pulled together and then shaken for 3 h at 30 °C with 533 µL lysozyme (100 mg/mL in DEPC water, Sigma Aldrich) and 7.7 µL β-mercaptoethanol (Sigma-Aldrich). Samples were frozen at − 20 °C, thawed and centrifuged at 4 °C for 20 min at 14.000 rpm. The DNA was extracted using the DNeasy blood and tissue kit (Qiagen) following the manufacturer’s instructions. DNA was quantified by measurements with both Nanodrop and Qubit (Thermofisher). DNA concentrations measured with the two instruments ranged from 18.6 to 48.6 ng/µL (Nanodrop) and from 13.7 to 48.8 ng/µL (Qubit). Purified DNA samples were subjected to 16S sequencing and shotgun analysis.

### 16S and Shotgun Sample Preparation and Sequencing

16S amplicons and total community genomic DNA were sequenced at Cemet GmbH (Tubingen, Germany). 16S V3–V4 amplicons were generated from 10 ng of DNA using forward primer S-D-Bact-0341-b-S-17 (5′-CCTACGGGNGGCWGCAG-3′) and reverse primer S-D-Bact-0785-a-A-21 (5′-GACTACHVGGGTATCTAATCC-3′) [35]. Only for the 2016 16S samples, two independent extractions and amplifications were carried out to explore variability of technical replicates. Library generation was performed according to the recommendations given by Illumina. Amplicons were sequenced as 2 × 250 bp read pairs on an Illumina Miseq instrument using MiSeq Reagent Kit v2. A minimum of 22,000 read pairs were generated per 16S samples. Shotgun genomic data was generated as 2 × 100 bp read pairs (2016 dataset) and 2 × 150 bp read pairs (2017 dataset) on an Illumina HiSeq instrument. An overview on shotgun read numbers and filtering is provided in Supplementary Online resource 1: Supplemental Table S1. Library preparation was performed according to the Illumina Nextera XT protocol.

### 16S and Shotgun Data Analysis

#### 16S Data

For the 16S data, after removal of primers, the read pairs were initially filtered by trimmomatic v0.38 [36] applying a minimum length-cutoff of 245 bp and a minimum average (Avg) quality values of 30. Read pairs were then combined into complete amplicon using FLASH v2.0 [37]. The resulting merged read pairs were then filtered omitting sequences shorter than 350 nucleotides. Average overall length of merged read pairs was 413.5 (median 412), minimum 351 and maximum 480 (Online resource 1: Supplemental Table S2).

16S amplicons were then rarefied to 22,000 merged read pair sequences using usearch [38] and clustered into operational taxonomy unit (OTUs) at 97% sequence identity. Taxonomic assignment was then performed using the usearch SINTAX algorithm [39] against the GTDB [40] release95 ssRNA archaea and bacteria database (https://data.ace.uq.edu.au/public/gtdb/data/releases/release95/95.0/). OTUs clustering and heatmaps were generated using the heatmap.2 function in ggplot2 (CRAN repository: https://cran.r-project.org/web/packages/ggplot2/index.html) from the R language (https://www.r-project.org/foundation/). Clustering was performed with heatmap.2 default parameters (complete agglomeration using the Euclidean measure to obtain a distance matrix). For bubble plots displaying the relative abundance of the 16S amplicons from the 2016 samples, values were averaged across the two replicates.

#### Shotgun Data

Shotgun read pairs were filtered with trimmomatic v0.38 [36] applying a minimum length-cutoff of 90 bp (2016 dataset) and 140 bp (2017 dataset) and a minimum Avg quality value of 28. Reads were filtered by trimmomatic as pairs in order to allow also running Kraken2 [41] for taxonomic binning in paired read mode using a pre-compiled version Kraken2 format of the GTDB database available at https://bridges.monash.edu/projects/Metagenomics_Index_Correction/65534. Assembly of shotgun read pairs into contigs was performed with MegaHit v1.2.9 using default parameters [42]. For each sample, the set of contigs obtained by MegaHit with length ≥ 5000 was confronted with a BLASTN database containing contigs from all other samples. Examination of the BLASTN search results was performed filtering out matches with ≥ 99% sequence identity and with a match length covering at least 50% of the query contig. MegaHit reports a multiplicity value for each contig representing the average coverage by reads for each contig. In order to normalize for the total number of read pairs in each sample, multiplicity values of the BLASTN hits selected with the above criteria were adjusted accordingly. All contigs obtained were also confronted with a BLASTN database containing all GTDB genomes filtering out matches with at least 90% identity over 10,000 nucleotides.

Functional annotation of shotgun reads was performed, due to limited computing resources, on 2 million randomly selected reads from each sample using DIAMOND [43] and the bacterial subsection of the NCBI nr database. Results from DIAMOND were then imported into MEGAN6 CE [44] and analysed using the SEED database [45]. In particular, variations in relative abundances associated to general pathways or to specific metabolic functions have been investigated using the SEED subsystems level 1 and level 2, respectively. Level 1 subsystems describe a specific biological process (similar to a pathway) or structural complex while level 2 subsystems describe the individual components of the level 1 process.

Reliability of read pair assignment to the *Lyngbya robusta* species by Kraken2 was estimated in the following way: the *Lyngbya robusta* (*Limnoraphis robusta* CS-951) genome assembly GCF_000972705.2 (ASM97270v2) was downloaded from NCBI, and the genome assembly contigs were merged into one contiguous sequence (7,314,117 nucleotides) and converted into a BLASTN database. Sequences of read pairs that had been assigned to *Lyngbya robusta* in the Kraken2 output files were extracted and assembled with MegaHit. The resulting contigs were then confronted with the BLASTN *Lyngbya robusta* database*.* A custom-written perl script then filtered the BLASTN output accepting, for each match start position, only the hit with the highest percent identity. In addition, the script also collected all regions of the merged *Lyngbya robusta* genome assembly that were covered by hits from the MegaHit contigs.

To prepare contigs for a MetaBat2 [46] analysis, all shotgun read pairs from the 2017 E samples were assembled with MegaHit and read pairs from the individual samples were aligned to the assembled contigs using bowtie2 [47]. Resulting bam files were then processed with MetaBat2 obtaining binned contigs. The same procedure (bowtie2 mapping and MetaBat2 binning) was then applied also to the shotgun data from the 2017 S and M samples and separately, to all 2016 samples. MegaHit analysis of all 2016 and 2017 (or only 2017) shotgun samples together was not possible due to limiting (memory) computing resources. Details of all MetaBat2 are available in Online resource 2: file vareselake_2.xlsx. N50 values provided indicate the length of that contig within the bin where about 50% of the complete bin length is covered, after ordering the contigs from longest to shortest. Assignment of MetaBat2 bins to known GTDB genomes/genome assemblies was then performed with BLASTN accepting hits of at least 1500 nucleotides with at least 90% identity. Completeness of MetaBat2 bins was performed with checkm [48] and further annotation of bins was performed with GTDBtk [49]. Estimate of phage content of the MetaBat2 bins was done with virsorter2 [50].

Network co-occurrence was performed with Cytoscape [51] and Conet [52] using the Kraken2 results from the 2017 shotgun data at the genus level.

All processing was performed on a workstation with one Xeon CPU (8 hyper-threads) and 48 Gb RAM.

### Redundancy Analysis

The Canoco 5 version [53] was used to run the analysis. Data were compositional with a gradient 2.1 SD units long, so linear method was preferred. Data from 16S sequencing were log-transformed and the taxa which relative abundance was > 1% were selected. Both axes were significant when *p* < 0.05.

## Results

### Physico-Chemical and Inorganic Chemical Parameters

In 2016 and 2017, six and eight sampling campaigns were performed at Lake Varese, respectively. During the 2016 study period, the 2.5 × Secchi depth (S), which is a good proxy to estimate the euphotic depth, varied between 7.6 and 13 m (see Table 1). Range and Avg of the physico-chemical parameters measured during the sampling campaigns (WT, CD, DO, OS, PAR, ORP and pH) are reported in Table 1.

**Table 1:** Range and average (Avg) of physico-chemical and biological parameters. *E*, Epi depth; *S*, 2.5 × Secchi depth; *M*, Meso depth; *Min*, minimum; *Max*, maximum; *SD*, standard deviation. (*) S depth: 13 m on 31/8/2016; 7.6 m on 7/9/2016; 8 m on 14/9/2016; 7.8 m on 21/9/2016; 10.3 m on 28/9/2016 and 13 m on 5/10/2016; 2 m on 2/8/2017; 6 m on 9/8/2017; 5 m on 23/8/2017 and 30/8/2017; 8.75 m on 27/9/2017 and 11 m on 4/10/2017

			E (0.5 m)				S (*)				M (13 m)			
	Parameters and units of measurement		Min	Max	Avg	SD	Min	Max	Avg	SD	Min	Max	Avg	SD
2016	Water temperature (°C)	(WT)	20.4	25.2	22.7	1.9	9.2	16.8	13.2	3.0	9.2	10.3	9.8	0.3
	Conductivity (µS/cm at 25 °C)	(CD)	242	252	246.5	3.7	307	319	312.8	4.0	312	323	318.3	3.1
	Dissolved oxygen (mg/L)	(DO)	8	9.7	8.6	0.5	0	1.1	0.2	0.4	0	0	0	0
	Oxygen saturation (%)	(OS)	91.2	120.7	102.7	8.1	0	10.8	2.1	4.1	0	0	0	0
	Photosynthetically active radiation (µE/m^2^/s)	(PAR)	74	2444	691.4	870.1	6	13	8	2.7	6	6	6	0
	Oxidation reduction Potential (mV)	(ORP)	− 51	206	130.1	100.8	− 154	212	41.3	156.1	− 175	− 136	− 152.7	12.5
	pH	pH	8.3	8.5	8.4	0.1	7.1	7.5	7.3	0.1	7	7.2	7.1	0.1
	Chlorophyll *a* (µg/L)	(Chl*a*)	1.5	12.8	5.8	3.9	1.6	15.4	8.3	5.9	0.8	4.7	2.9	1.6
2017	Water Temperature (°C)	(WT)	19.5	27.2	23.5	3.1	10.8	26.4	20.1	5.8	9.2	9.8	9.3	0.4
	Conductivity (µS/cm at 25 °C)	(CD)	172	206	188.8	11.3	208	281	245.5	27.9	281	312	293.5	22.5
	Dissolved oxygen (mg/L)	(DO)	8.3	11.1	9.8	1.1	0.3	11.2	3.9	3.9	0.2	0.5	0.3	0.1
	Oxygen saturation (%)	(OS)	102.6	136.5	116.8	11.6	2.4	139.5	47.1	47.6	1.7	4.5	2.4	0.6
	Photosynthetically active radiation (µE/m^2^/s)	(PAR)	141	443	272.6	125.0	6	235.9	25.2	56.4	0	6	5.6	1.5
	Oxidation reduction potential (mV)	(ORP)	128	284	204.9	64.9	− 10	273	172.7	110.1	− 165	− 39	− 115.3	57.8
	pH	pH	8.6	9.1	8.6	0.5	7.3	8.9	7.5	0.8	5.6	7.5	6.9	0.7
	Chlorophyll *a* (µg/L)	(Chl*a*)	4.1	19.2	12.1	4.8	7.1	22.4	13.6	5.5	2.4	22	5.9	6.3

Generally, a uniform physico-chemical pattern was observed across all three depths during the sampling period. At the greatest depth (M), WT showed lower values compared to an Avg temperature of 22.7 °C recorded at the surface (E) and the CD was minimum at the surface (242 µS/cm at 25 °C) and maximum at the M depth (323 µS/cm at 25 °C). In the selected study period, the water column was stratified as evidenced by the DO levels measured at the different depths. The OS was observed to decrease along the water column, with values always equal to 0% at the M depth and PAR showed a fluctuation particularly at E depth (Online resource 1: Supplemental Table S3). The pH was generally alkaline with highest values around 8 measured at E depth. Similar values for physico-chemical parameters were observed in 2017 (see Table 1 and Online resource 1: Supplemental Table S4). No measurements were performed on 19/7/2017 and 26/7/2017 due to technical problems with the probe. Nutrients were also measured in Lake Varese: levels for nitrate were below the LOD (LOD = 0.049 mg/L) in most of the samples analysed, ammonia was less present in the surface but more abundant in the anoxic bottom (0.3–0.8 mg/L) and, in September 2016, total P ranged from 0.06 to 0.26 mg/L at E depth (Online resource 1: Supplemental Table S5).

### Chlorophyll a and Cyanotoxins Analysis

**Fig. 2:**
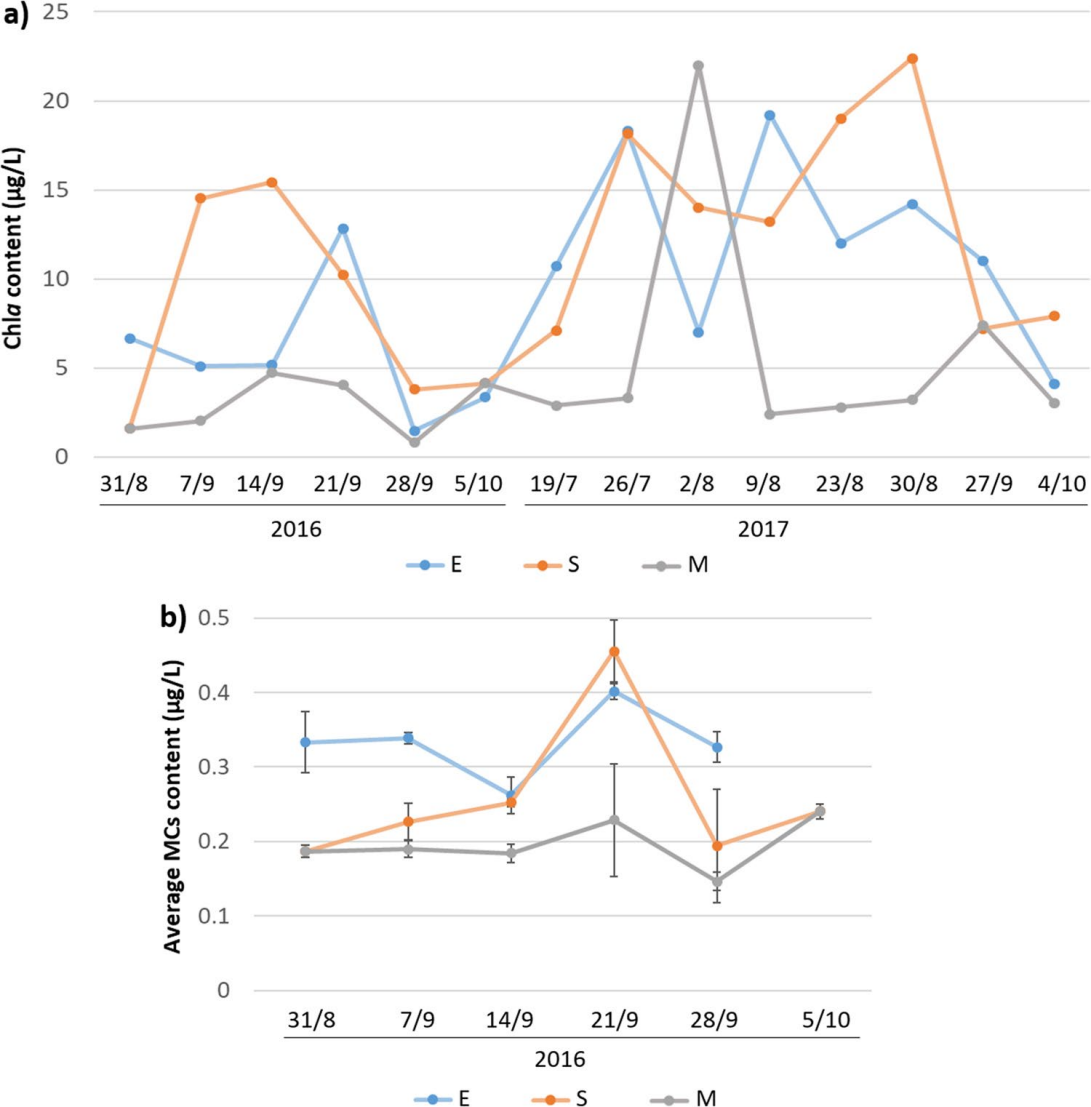
Chlorophyll *a* (Chl*a*) and total Microcystins (MCs) content in raw samples collected in Lake Varese. **a** Chl*a* concentrations, in µg/L, measured during the sampling dates (*x* axis) at the surface (E), 2.5 × Secchi disk depth (S) and 13 m depth (M). **b** Total MCs content measured by ELISA and detected across the study period (dates reported on the *x* axis) at the surface (E), 2.5 × Secchi disk depth (S) and 13 m depth (M). Results are expressed as means of three replicates and standard deviation bars are indicated for each sampling point

Chl*a* concentration was found to be generally higher in both E and S than M depth (Fig. 2a, Table 1 and Online resource 1: Supplemental Table S6). In 2016, the highest Chl*a* levels, exceeding the value of 14 µg/L, were observed in S samples, while Chl*a* analysis at E depth revealed a peak value close to 13 µg/L on the 21st of September (Fig. 2a and Online resource 1: Supplemental Table S6). During the 2017 campaign, Chl*a* content observed in the epilimnion was alternately higher or lower than the concentrations detected at S depth (Fig. 2a and Online resource 1: Supplemental Table S7). Peak values were reported on 2/8/2017 (M), 9/8/2017 (E) and 30/8/2017 (S) (Fig. 2a and Online resource 1: Supplemental Table S7).

In 2016, levels of the cyanotoxins MCs, SX and AX were measured by ELISA in raw water samples, revealing the intra- and extracellular amount that was present in water at the time of sampling. MCs content exceeded the value of 0.35 µg/L only on the 21st of September in both E (0.40 µg/L) and S samples (0.46 µg/L) (Fig. 2b). In all other sampling dates, MCs concentration was higher in E samples than in M and S samples, with values ranging from 0.26 to 0.40 µg/L (Fig. 2b).

SX concentration was below the detectable level (< 0.020 µg/L) in most of the samples analysed (data not shown). A low SX content was detected in only two samples found positive and corresponding to the E samples collected on 14th (0.021 µg/L) and 28th of September (0.020 µg/L). No AX levels were detected during the campaigns (LOD = 0.1 µg/L, data not shown).

### 16S Analysis

#### Community Composition of Main Phyla

Microbial community composition was analysed by 16S sequencing in water samples collected during the two sampling campaigns (2016 and 2017) at the three different depths: E (0.5 m), S and M (13 m) (the M depth corresponded to the S depth on 31/8/2016 and 5/10/2016). To assess the variation in the microbial community in Lake Varese, a first global and unbiased view was obtained by clustering OTUs [54] counts (Online resource 1: Supplemental Fig. S1) from samples collected during the two sampling campaigns. Four main clusters were readily visible: cluster 1 present in all samples, clusters 2 and 4 present in M and some S samples and cluster 3 present in E and S samples (Online resource 1: Supplemental Fig. S1). A closer inspection of the taxonomies (phylum level) present in each cluster did however not reveal dominant phyla. In addition, the analysis also indicated the high reproducibility of replicate samples collected during 2016.

Taxonomic binning was then obtained by randomly choosing 22,000 amplicons from each sample, clustering amplicons into OTUs at 97% sequence identity and assigning taxonomies through application of the usearch Sintax algorithm [39]. Rarefaction analysis of OTUs indicated an essentially complete overview on the community complexity for all the samples (Online resource 1: Supplemental Fig. S2). Highest diversity was present in the 2016 M samples, while 2017 E samples showed lowest complexity (Online resource 1: Supplemental Fig. S2).

#### Microbial Community Structure Based on 16S Amplicons

Between 84 and 95% of OTUs were successfully assigned at the phylum level, revealing an overall relatively constant profile, with some fluctuations across samples and seasons. Taxonomic analysis at phylum level showed that, in most of the samples, Proteobacteria was the predominant taxonomic group followed by Actinobacteriota, Cyanobacteria and Bacteroidota (Online resource 1: Supplemental Fig. S3). A unique peak of Bdellovibrionota, a bacterial predator present in lakes [55], was found in one of the 2016 S samples (Online resource 1: Supplemental Fig. S3). Proteobacteria dominated the profile in all E and M samples collected in both 2016 and 2017. In the dataset 2016, Cyanobacteria showed lower abundance in M samples compared to the other two depths and were found to be the most abundant community in S samples collected during three sampling campaigns in September 2016 (7/9/2016, 14/9/2016 and 28/9/2016) (Online resource 1: Supplemental Fig. S3). In the same month, the cyanobacterial relative abundance at E depth exceeded the value recorded at S depth only on the 21st of September. That abundance was found to be above all the cyanobacterial content observed in 2016 at E depth. Metagenomic data from 2017 suggested that Lake Varese experienced a continuous cyanobacterial bloom during the study period, as also confirmed by the visual inspection. In datasets 2016 and 2017, the phylum Desulfobacterota was identified at percentages above 1% in all 2016 M samples and in three S samples (28/9/2016, 9/8/2017 and 4/10/2017) (Online resource 1: Supplemental Fig. S3).

**Fig. 3:**
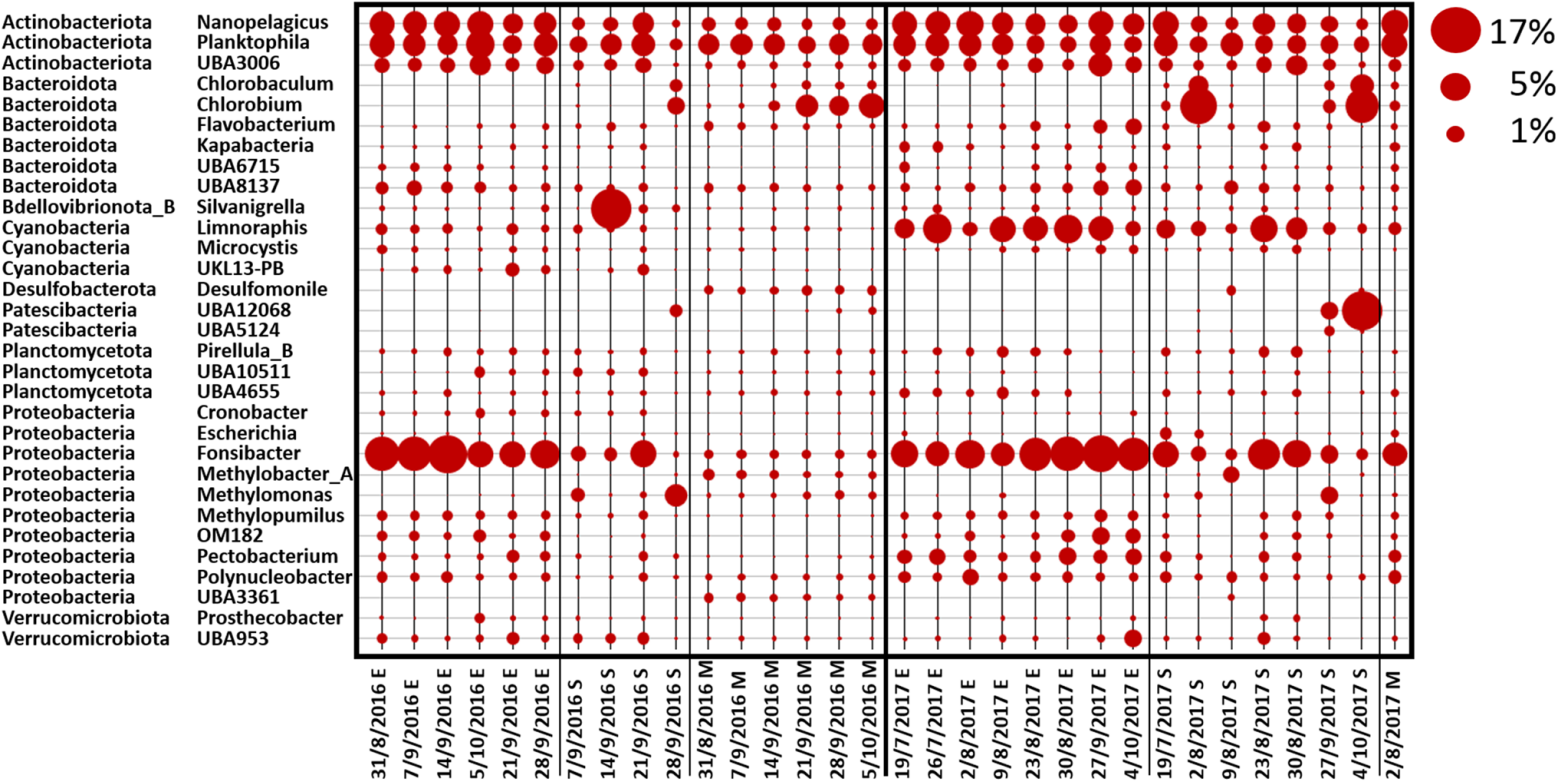
Community composition at the genus level as determined from the 16S data. The community composition is shown for all the water samples collected during the sampling period in Lake Varese at the three different depths (Epi (E), 0.5 m; 2.5 × Secchi (S) and Meso (M), 13 m). Samples were sequenced for 16S rRNA (V3–V4 region) and analysed at the genus level. Secchi depths were measured at each site using a Secchi disk and values multiplied by 2.5. The M depth corresponded to the S depth on 31/8/2016 and 5/10/2016. For 2016 replicate samples, the average value is shown. Circles shown represent percent values within the fraction of taxonomy assigned shotgun sequences (circles shown at the top right of the figure represent example percent values)

At the genus level, the fraction of OTUs assigned to a specific taxonomy varied considerably, with values from only 20 up to 60%, thus providing only a partial snapshot of a more detailed taxonomic composition. As expected, fluctuations in the profile were much more pronounced compared to data at phylum level, both between seasons and between samples obtained at different depths. *Planktophila*, *Fonsibacter* and *Nanopelagicus*, three of the most ubiquitous and abundant freshwater bacterial genera, showed great variability at the three different depths with E samples usually showing the highest levels detected among all samples (Fig. 3). Analyses at the genus level allowed to more precisely characterize the unique peak of Bdellovibrionota observed at the phylum level in the dataset 2016 (Supplemental Fig. S3), as belonging to the genera *Silvanigrella* (Fig. 3), according to the GTDB database classification. Microcystis was detected at very low levels in all samples (Fig. 3). In this study, the cyanobacterial phylum was mainly represented by the genus *Limnoraphis* (also known as *Lyngbya)*, which is described to form blooms in freshwater environments [56, 57] (Fig. 3). Methanotrophs and methylotrophs like *Methylomonas*, *Methylobacter* and *Methylopumilus* were also detected (Fig. 3).

**Fig. 4:**
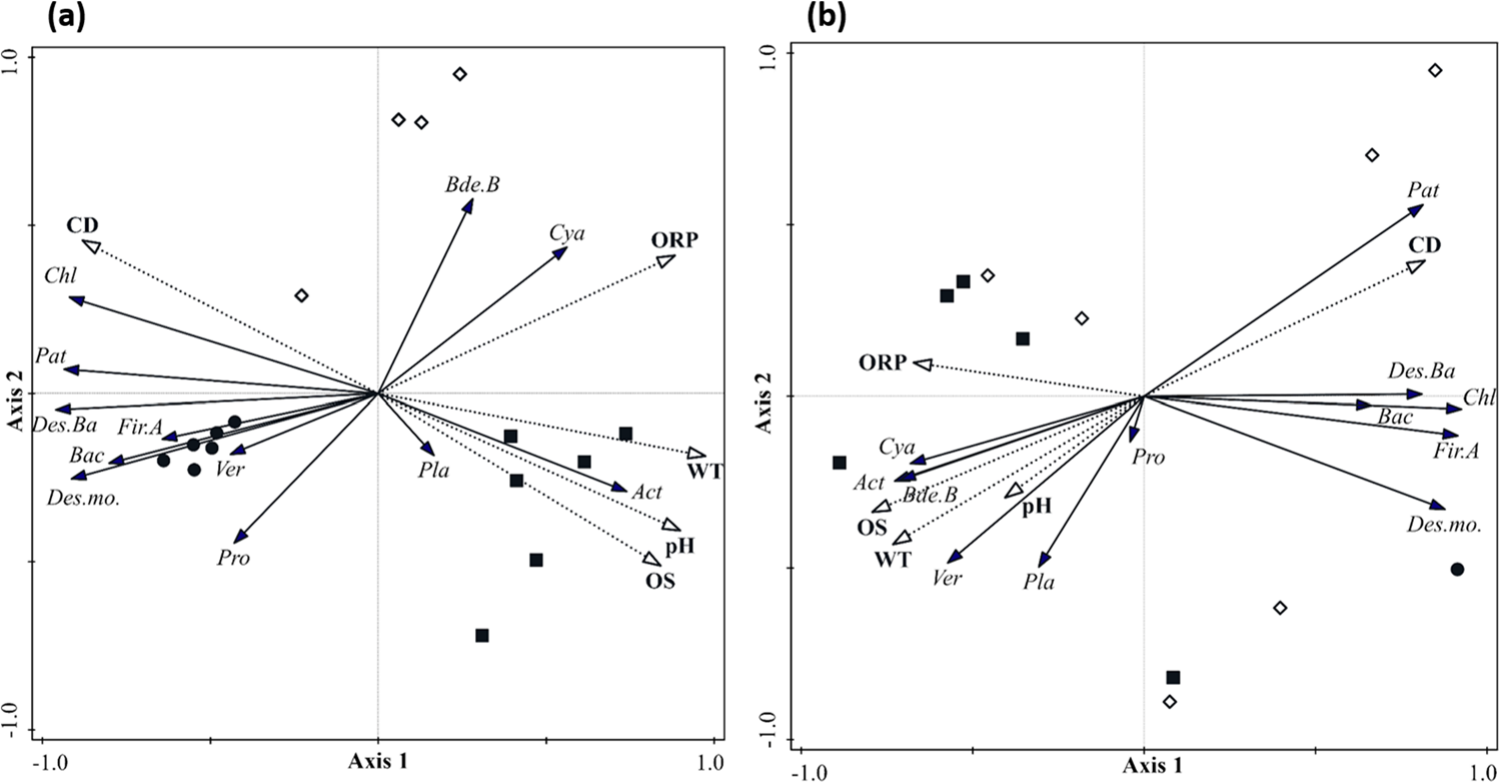
Redundancy analysis (RDA) of the 16S data 2016 and 2017 at phylum level. RDA triplot with 16S taxa (solid lines) with abundance > 1%, environmental variables (dotted lines) and samples collected from August 31st, 2016, to October 5th, 2016 **(a)**, and from August 2nd, 2017, to October 4th, 2017 **(b)**. Black circles are samples from the Meso layers, diamonds are samples from the 2.5 × Secchi layers, black squares are samples from the Epi layers. Abbreviations of environmental variables are as follow: pH, conductivity (CD), oxidation reduction potential (ORP), oxygen saturation (OS) and water temperature (WT). Abbreviations of taxa are as follow: Actinobacteriota (Act), Bacteroidota (Bac), Bdellovibrionota_B (Bde.B), Chloroflexota (Chl), Cyanobacteria (Cya), Desulfobacterota (Des.Ba), Desulfuromonadota (Des.mo.), Firmicutes_A (Fir.A), Patescibacteria (Pat), Planctomycetota (Pla), Proteobacteria (Pro), Verrucomicrobiota (Ver)

To verify how environmental variables affect taxa distribution derived from 16S sequencing analysis, a redundancy analysis (RDA) was carried out (Fig. 4). RDA summarizes part of the variation in taxa composition explained by environmental variables. The first two axes in both RDAs were significant (*p* < 0.05) and they explained 80% of cumulative variations and 97% of fitted cumulative variation. It means that the selected environmental variables well explain the species distribution.

Surface water layers (E and S) were characterized by higher temperature, pH, ORP and OS than the M layer, where only CD was high. These parameters all correlated well with the positive side of the first axis in 2016 and with the negative side of the first axis in 2017. Most of the taxa were significantly correlated with the environmental parameters, either directly or indirectly. Only Planctomycetota and Verrucomicrobiota in 2016, and Proteobacteria in 2017 did not have significant correlations.

### Shotgun Metagenomics

#### Microbial Community Structure Based on Shotgun Data

Using the Kraken2 algorithm in combination with the GTDB release89 database, between 14 and 29% of the shotgun reads, analysed as read pairs, could be classified at the phylum level, reproducing relatively well the profile obtained by the 16S data. Supplemental Fig. S4 (Online resource 1) shows the phylum level analysis of the shotgun reads. Shown values represent, for each taxa, the relative abundance (percentage) with respect to all shotgun reads for which a phylum level classification had been obtained. In the E samples, the 2016 results indicate highest levels of Cyanobacteria on 14/9/2016 and 21/9/2016 (in the 16S data, the peak at E depth was on 21/9/2016, Online resource 1: Supplemental Fig. S3), while for S samples, a peak in cyanobacterial content was registered on 14/9/2016 (Online resource 1: Supplemental Fig. S4) and shotgun data confirmed the low levels of Cyanobacteria in the M region. Like for the 16S data, in 2017, the cyanobacterial abundance in the epilimnion suggested a continuous bloom from July to August, showing a drop starting from September as suggested from visual inspection. The low presence of Cyanobacteria at the M depth was also confirmed in 2017. In both the 2016 and 2017 dataset, some variations of the overall microbial composition were also observed for Proteobacteria and Actinobacteriota (Online resource 1: Supplemental Fig. S4). The absence of the *Bdellovibrionota* peak reported in 16S analysis could be due to the fact that a partial *Silvanigrella* genome assembly (GCA_014281055 NCBI Genome database) has only been generated very recently.

**Fig. 5:**
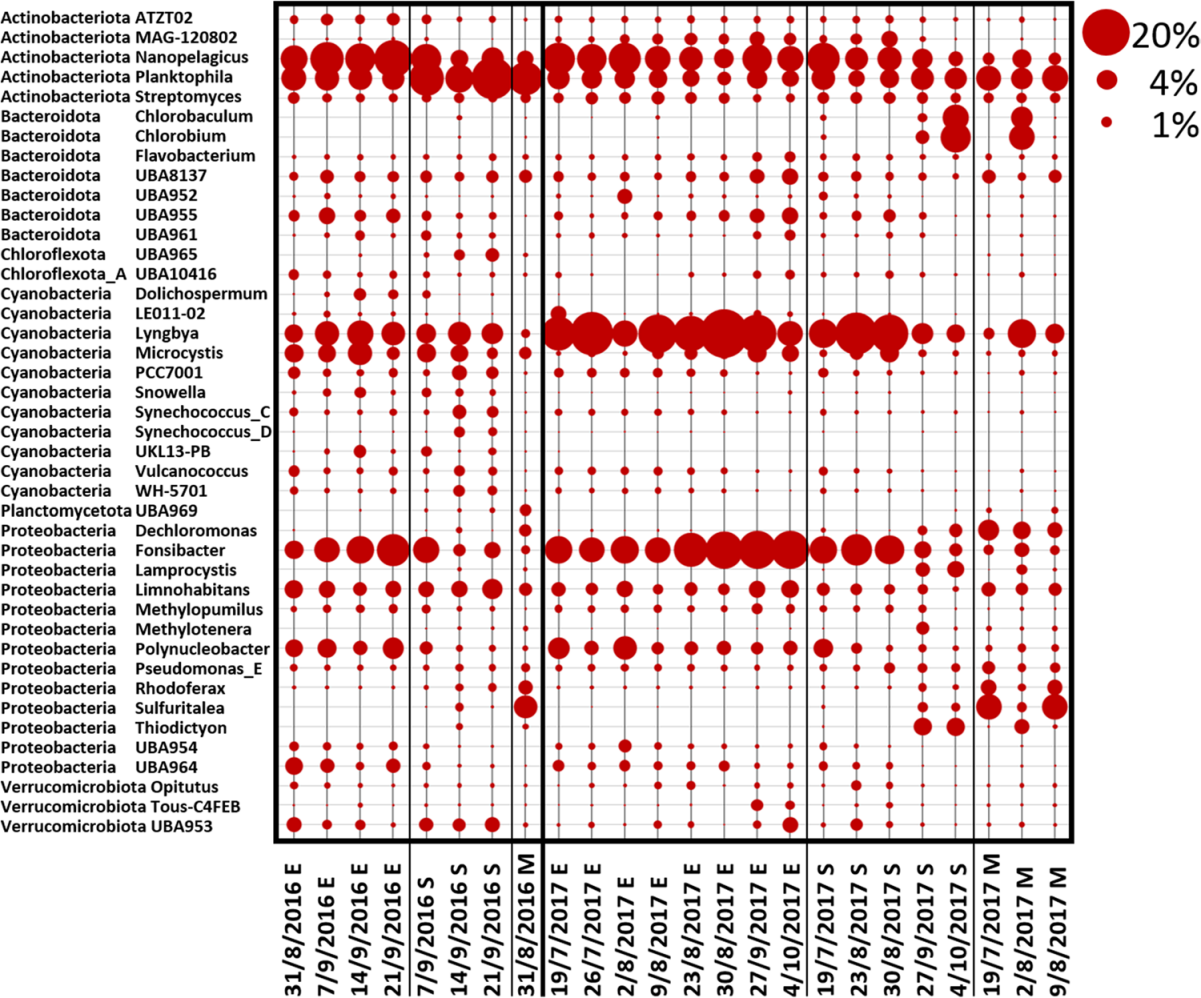
Community composition at the genus level as determined from the shotgun data. The community composition is shown for all water samples collected during the sampling period in Lake Varese at the three different depths (Epi (E), 0.5 m; 2.5 × Secchi (S) and Meso (M), 13 m). Samples were analysed for shotgun sequencing. The figure shows variations in the microbial community at the genus level. Secchi depths were measured at each site using a Secchi disk and values multiplied by 2.5. The M depth corresponded to the S depth on 31/8/2016. Circles shown represent percent values within the fraction of taxonomy assigned shotgun sequences (circles shown at the top right of the figure represent example percent values)

**Fig. 6:**
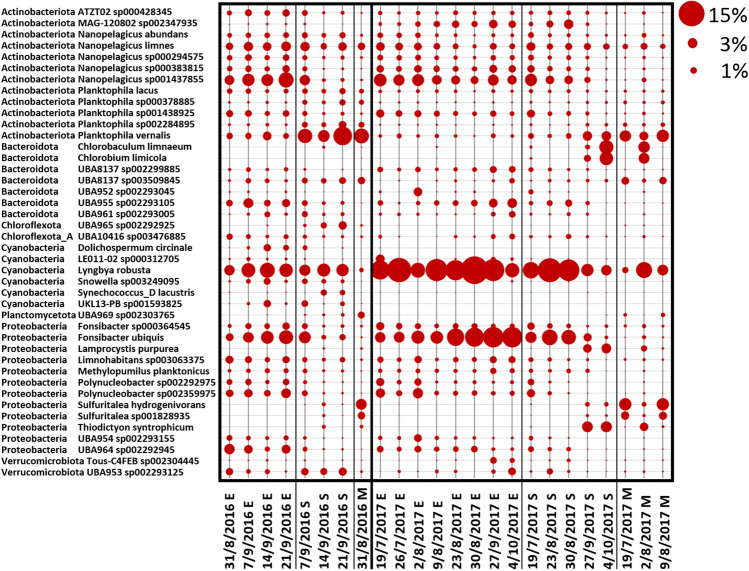
Community composition at the species level as determined from the shotgun data. The figure shows variations in the microbial community at the species level. For details, refer to the legend of Fig. 5

At the genus and species level, only a small fraction of shotgun reads, less than 5% could be classified thus allowing only a very limited view into the complexity of the bacterial community at this level. However, at least at the qualitative level, some additional information was extracted. *Lyngbya* was the dominant cyanobacterial genera with *Lyngbya robusta* (*Limnoraphis robusta*) being the most abundant species (Fig. 5 and Fig. 6). This species was already observed in other lake bloom events [58, 59]. *Microcystis* was generally present at low level in 2017 (Fig. 5) and genera like *Synechococcus* and *Snowella* were also detected in our samples. *Planktophila*, *Fonsibacter* and *Nanopelagicus* were among the most abundant bacterial genera detected at E depth across all samples (Fig. 5). Analyses at species level revealed that the occurrence of these genera was mainly attributed to the species *Planktophila vernalis*, *Fonsibacter ubiquis* and *Nanopelagicus sp001437855* (Fig. 6). Sequences corresponding to *Sulfuritalea hydrogenivorans*, a sulphur-oxidizing species previously isolated from stratified freshwater lakes [60], were found to be generally greater in the mesolimnion compared to the other depths (Fig. 5 and Fig. 6). OTUs belonging to *Thiodictyon syntrophicum* [61], another species involved in sulphur oxidation, were also identified in both M and S samples (Fig. 5 and Fig. 6), as well as *Chlorobium* which is a photoautotrophic sulphur oxidizer (Fig. 5 and Fig. 6). This is consistent with the negative ORP values and anoxia at the bottom of the lake (Online resource 1: Supplemental Tables S3 and S4) which could allow the biological production of reduced species of sulphur from oxidized species as sulphate (Online resource 1: Supplemental Table S5), available for the metabolism of autotrophic sulphur oxidizers.

To complement the taxonomy-focused Kraken2 analysis, shotgun read pairs from all individual samples were submitted to MegaHit for contig assembly. Maximal length and total number of the assembled contigs varied considerably between samples (maximal length from 42,318 to 821,984, number of contigs from 63,170 to 475,171) (Online resource 1: Supplemental Table S8). Presence of highly similar contigs (longer than 5000 nucleotides, 99% sequence identity, match longer than 50% of query length) across all samples was then examined with BLAST searches (Online resource 1: Supplemental Table S9). Putative assignment of contigs to known GTDB genomes was also performed through a BLAST search (Online resource 1: Supplemental Table S10). Contigs matching the *Lyngbya robusta* genome (*Limnoraphis robusta CS-951*; NZ_LATL02000001.1; assembly GCF_000972705.2) were detected in many samples with the highest estimated abundance (MegaHit contig coverage) in the 30/8/2017 E sample, like in the Kraken2 analysis (Online resource 1: Supplemental Table S9). For the 2016 samples, with a lower number of read pairs, no contig with length > 10,000 nucleotides and a sequence similarity higher than 90% to *Lyngbya robusta* was detected.

An additional analysis was then performed with all read pair sequences that were assigned to *Lyngbya robusta* by Kraken2. The read sequences were subjected to a MegaHit assembly run and the resulting contigs (Online resource 1: Supplemental Table S11; 2124 contigs with length 200 to 78,260, N50 14,006 and total length 7,208,187 nucleotides) were confronted with the GTDB genome database. A large portion of the contigs matched to *Lyngbya robusta* assembly as the best hit, with generally high sequence identity values (Online resource 1: Supplemental Table S11). All these results indicated that the Kraken2 taxonomy assignment, although most likely not perfect and less reliable at the lower ranks, had correctly identified a *Lyngbya* species closely related to *Lyngbya robusta.*

Finally, shotgun read pairs from the 2017 E, 2017 M and S and from the 2016 E, M and S samples were analysed with MetaBat2. Out of the 114 bins obtained for the 2017 E samples, only a small portion (total of 30) could be related to GTDB genomes (Online resource 1: Supplemental Table S12). The best annotated bin corresponded again to *Lyngbya robusta* (*Limnoraphis robusta*) with an estimated completeness of 98% (obtained by checkm) and an overall nucleotide identity of 96.35% determined by GTDBtk (Online resource 2). Instead, for the 2016 samples, *Lyngbya robusta* (*Limnoraphis robusta*) was present only with low relative abundance (Online resource 1: Supplemental Table S13 and Online resource 2).

A final analysis using virsorter2 revealed that a large portion of the sample metagenomes, in particular MetaBat2 bins with a relatively small overall length, are represented by phages (Online resource 1: Supplemental Tables S12 and S13), often corresponding to MetaBat2 bins with the highest relative abundance values. Complete results are available in Online resource 2. However, comparison of the “phage bins” with known phage sequences using DIAMOND did not allow to more precisely identify their taxonomy (Online resource 2).

#### Functional Profile

Functional profiling of the shotgun samples, obtained by a DIAMOND blastx search against the bacterial subsection of the NCBI nr database and MEGAN6 mapping to the SEED database [62], showed, at the SEED level 1, an overall rather constant profile during the 2016 and 2017 seasons (Online resource 1: Supplemental Fig. S5).

**Fig. 7:**
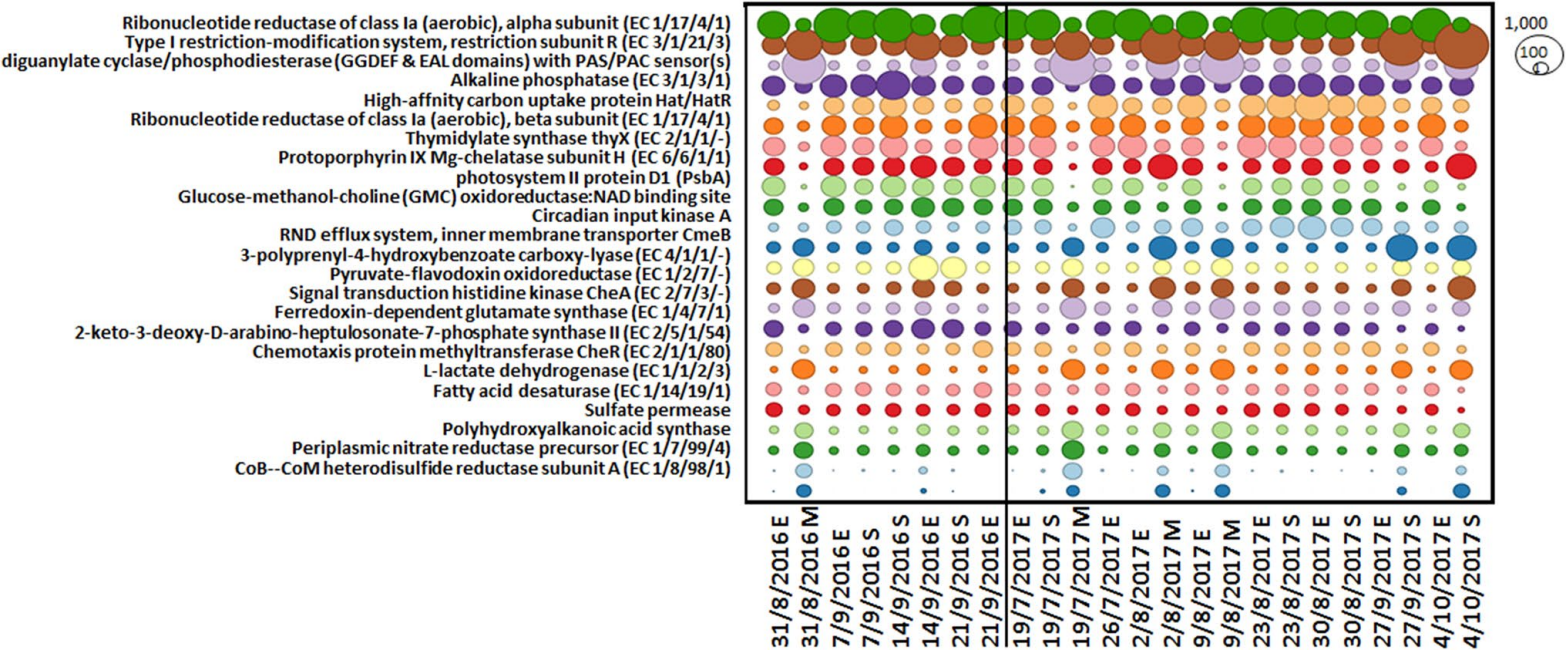
Functional profile (SEED level 2) of the microbial community (shotgun samples). A representation of the SEED level 2 functional profiles as determined by MEGAN6 from DIAMOND blastx searches. Circles shown are proportional to the number of matches against the indicated SEED level 2 class. The figure shows the SEED functions with more than 2000 matches across all samples and with an at least threefold difference between the maximum and minimum values observed. E stands for Epi, M for Meso and S for 2.5 × Secchi

Instead, changes in the composition of metabolic functions occurred at the SEED level 2 (Fig. 7). In particular, the low cyanobacterial levels observed at the M depths (Online resource 1: Supplemental Fig. S4) appear to be associated with a distinct functional profile compared to samples collected at E and S depths. Only two S samples (27/9/2017 and 4/10/2017) followed a similar pattern as M samples, probably due to the high sampling depth (8.75 and 11 m, respectively). Samples collected at E depth showed no marked differences in their functional profiles compared to most of the S samples (Fig. 7).

Looking at the M pattern, most of the metabolic functions were little represented at this water depth compared to the E and S regions. One abundant function at M depth, less pronounced compared to the other profiles, involves diguanylate cyclase phosphodiesterase (DGC-PDE) domains (GGDEF & EAL) comprising a PAS/PAC sensor. Previous studies have described the role of DGC’s in cyclic dimeric GMP (c-di-GMP) synthesis [63], a second messenger implicated in regulating different processes in bacteria (e.g. biofilm formation and virulence etc.) and its hydrolysis is mediated by phosphodiesterase (PDEs) [63]. In Cyanobacteria, high levels of intracellular c-di-GMP have been associated to a reduced cellular buoyancy [64]. Tuckerman et al. described a DGC protein controlled by oxygen in *Escherichia coli* [65]. Oxygen, as a pivotal ligand, was also found in *Acetobacter xylinum* by Chang et al. [66] as a switch off for cellulose synthesis in biofilm production as well as in the pathogen *Bordetella pertussis* with a similar function [67]. It is likely that the modulated expression of the DGC/PDE activity observed at the different water depths influences the cyanobacterial distribution in the water column or the activation of biofilm production in an oxygen scarce environment such as the M depth. Only a few reads matched genes related to the photosystem II process (e.g. photosystem II protein D1; see Fig. 7) and the highest relative abundance was identified in reads assigned to the periplasmic nitrate reductase (Nap) precursor, to the CoB-CoM heterodisulfide reductase subunit A (HdrA) and sulphate permease (SulP) activities. Nap is a protein expressed only in Proteobacteria [68], the predominant phylum at all depths, and its activity is associated to the reduction of nitrate [69–71], while the modulation in the CoB-CoM HdrA and SulP may indicate a role in methanogenesis and sulphate reduction activities, respectively [72].

Furthermore, examination of the *Lyngbya robusta* genome annotation revealed an extended region comprising nitrogenase and nitrogen-fixing genes conserved in the MetaBat2 bin closely related to *Lyngbya robusta* (Online resource 1: Supplemental Table S14).

## Discussion

The aim of this study was to investigate the microbial community composition in Lake Varese and temporal and spatial changes during the bloom events, captured in the sampling campaigns performed in 2016 and 2017. In 2016, metagenomics 16S analysis revealed that major differences in the bacterial community composition during the study period were concentrated more in the S depth area compared to the E and M zones. Cyanobacteria were highly abundant particularly in the surface layers (E and S depths), with low levels recorded in M samples, as confirmed as well by the RDA analysis (Fig. 4 and Online resource 1: Supplemental Fig. S3 and S4). Indeed, the visual inspection of the water during the sampling campaign indicated that the bloom was characterized by a green discoloration of the water due to large growth of cyanobacteria and it was very dense. These data would then suggest that Cyanobacteria were likely responsible for the bloom and the Chl*a* peak observed in September 2016 (Fig. 2a). However, a complementary 18S metagenomics sequencing and a microscopy analysis would be recommended in order to discard a possible contribution of phototrophic eukaryotes during blooms in 2016 and 2017. A complementary analysis of the shotgun data using plant sequence data did not reveal a significant presence of algae (data not shown).

Looking at the community composition, the 16S metagenomic analysis revealed that Actinobacteriota, Proteobacteria, Bacteroidota and Verrucomicrobiota were the four phyla also dominating the community profile at all the three depths (Online resource 1: Supplemental Fig. S3) in accordance with the reported presence of bacteria belonging to these phyla in the bloom community [73–77]. In 2017, oscillations in cyanobacterial abundance and their visual observation in water indicate that Lake Varese experienced a continuous cyanobacterial bloom until a reduction starting from September. The microbial community observed at the three water depths was dominated by the same phyla already detected in 2016 (Proteobacteria, Actinobacteriota, Cyanobacteria and Bacteroidota). 16S data showed that the cyanobacterial abundance in 2017 was confined to the epilimnion (Supplemental Fig. S3) and in the RDA analyses, cyanobacteria were more correlated to surface samples (E depth) showing higher values for the physico-chemical parameters OS, WT and pH in 2017 than in 2016.

Since only small changes in the microbial community population were observed at the M depth during the 2016 campaigns, only one sample was analysed for 16S at this depth in 2017, while three samples were selected for the shotgun analysis. Phylum-level classification of the shotgun dataset for 2016 and 2017 reproduced relatively well the profile obtained by the 16S data. The overall differences observed in the samples are not surprising since the two sequencing methods are based on different methodological approaches, which can influence the data analysis. Although only a small fraction of shotgun reads was annotated at the genus and species level, *Lyngbya robusta* was identified for the first time in Lake Varese, representing the dominant cyanobacterial species, in particular in the 2017 samples (Fig. 6), by direct taxonomic binning and through metagenomic contig assembly. Other species, mainly detected at E depth, included *Planktophila vernalis*, *Fonsibacter ubiquis* and *Nanopelagicus sp001437855* (Fig. 6). *Lyngbya robusta* is a species involved in nitrogen fixation [57, 78–80] and its blooms have been observed in freshwater waterbodies including Lake Atitlan (Guatemala) where concentrations of NO_3_^−^ and NH_4_^+^ were indicative of a N limitation, as also observed in Lake Varese, where these values were found to be below the LOD in most of the samples analysed (Online resource 1: Supplemental Table S5) [58]. While no reads assigned to the nitrogenase activity were detected by the SEED analysis (Fig. 7), examination of the *Lyngbya robusta* genome annotation revealed an extended region comprising nitrogenase and nitrogen-fixing genes conserved in the MetaBat2 bin closely related to *Lyngbya robusta* (Online resource 1: Supplemental Table S14). The heterotrophic bacteria identified in Lake Atitlan during these bloom phenomena included phyla also detected in Lake Varese such as Actinobacteria, Bacteroides, Proteobacteria and Firmicutes [79] whose putative role was mainly associated with an increased nitrogenase activity. In the same lake [79], analysis of the *nihF* gene profile indicated other potential nitrogen-fixing bacteria to be present, for example genus *Methylomonas*, also reported in Fig. 3. The nutrient content measured in Lake Varese could explain the competitive advantage of *Lyngbya robusta* over other cyanobacteria species such as *Microcystis*, a non-diazotrophic cyanobacteria which use N_2_-fixing cyanobacteria as a source of nitrogen [81]. The same mechanism could explain the presence in 2016/2017 samples of other non-diazotrophic cyanobacteria like *Synechococcus* and *Planktothrix*, which could be also dominant or co-dominant with nitrogen-fixing cyanobacteria [82–85]. Like these genera, *Microcystis* can be just as abundant as N_2_-fixing cyanobacteria under N-limited conditions [86, 87]. In the shotgun analysis, values of relative abundance observed on some dates in 2016 at E depth were similar between *Lyngbya* and *Microcystis*, while in 2017, very low levels of *Microcystis* were detected in most of the samples (Fig. 5). In the literature, there is no evidence showing that *Lyngbya robusta* is a MCs-producer cyanobacterium. Their production most likely involve the genus *Microcystis* which is a well-known producer of these toxins [1] and has been also detected in the shotgun data (Fig. 5). Other cyanobacteria like *Planktothrix* could contribute to the MCs production [1]. *Planktothrix* was previously identified in Lake Varese during a bloom event [30] and was also found in the shotgun analysis (at the genus and species level), although at very low levels of relative abundance (< 1%).

The functional profile of the microbial community showed that during the two annual campaigns, most of the E and S samples had a similar abundance of reads assigned to the different biological processes (Fig. 7). Lack of marked changes in functional genes was probably due to the limited variations in taxonomic composition of the bacterial community at the higher taxonomic ranks. However, a specific pattern was found to be associated with all M samples analysed and two S samples (27/9/2017 and 4/10/2017) collected at high depth (8.75 and 11 m, respectively). The functional profile identified in these samples may reflect a community composition characterized by low levels of Cyanobacteria. A low relative abundance of reads assigned to the photosystem II process (photosystem II protein D1; see Fig. 7) is notable in M and some S samples and can be explained by the low representativeness of Cyanobacteria at high water depths where light radiation is scarce.

The high relative abundance of genes involved in the Nap precursor activity (Fig. 7), which is associated to the reduction of nitrate during nitrate respiration [69–71], could be due to the selective presence of the Nap protein in Proteobacteria [68], a phylum highly present at M depth, and by the lack of oxygen detected in the mesolimnion (Table 1, Fig. 7, Online resource 1: Supplemental Tables S3 and S4, Supplemental Fig. S4). In addition, the presence of ammonia up to 0.831 mg/L in the M anoxic environment (Online resource 1: Supplemental Table S5) supports nitrate reduction. The relative abundance of reads assigned to the CoB-CoM HdrA activity was observed to be high at M depth (Fig. 7) and may indicate methanogenesis functions even though Archaea have not been identified through shotgun. Although Hdr has been detected only in methanogenes [72], homologous proteins show a widespread distribution and the “bacterial heterosulfide” Dsrc has been detected in *Desulfovibrio* spp. [88]. In this study, *Desulfovibrio* spp. (classified under the phylum Desulfobacterota) were found in high abundance at M depth (Online resource 1: Supplemental Fig. S3) where oxygen was absent, and negative values for the ORP were found (Online resource 1: Supplemental Table S3). Desulfobacterota are anaerobic bacteria which gain energy by sulphate reduction or sulphur disproportionation [89, 90]. Interestingly, an activity which is highly represented in all M samples and can be related to the sulphate reduction process involves the SulP family (Fig. 7), found to be active in Desulfobacterota [91]. In this functional mechanism, sulphate has to be imported inside the cell and then activated with adenosine triphosphate (ATP) in order to be reduced to sulphide by the sulphate respiratory pathway [92]. In the shotgun datasets, reads corresponding to sulphur-oxidizing bacteria, *Sulfuritalea hydrogenivorans*, *Thiodictyon syntrophicum* and *Chlorobium*, were also detected (Fig. 6). *Thiodictyon* spp. were already found throughout the anoxic zones of water column in a freshwater lake [93], and the strain *Sulfuritalea hydrogenivorans* sk43H was identified in stratified lakes [60]. *Sulfuritalea hydrogenivorans*, the major planktonic sulphur oxidizer detected at M depth, is facultatively anaerobic but an autotrophic growth is also observed under anoxic condition in which hydrogen, elemental sulphur and thiosulphate are used as electron donor, and nitrate as electron acceptor [94]. Moreover, the genus *Sulfuritalea* is known to express the *dsrA* gene encoding dissimilatory sulphite reductase [95]. We also detected methanotrophs and methylotrophs (*Methylomonas*, *Methylobacter* and *Methylopumilus*) in our samples, which are bacteria that use methane and single carbon compounds (i.e. methanol), respectively, as energy source [96].

Overall, the results of this study reveal the potential of high-throughput sequencing (HTS) in obtaining a snapshot of the bacterial community structure, only partially investigated during previous studies performed in Lake Varese, providing more details regarding the temporal and spatial modulations during bloom events. In particular, the HTS approach allowed to identify for the first time *Lyngbya robusta* as the cyanobacterial species mainly responsible for the bloom. Further analyses aimed at correlating the relative abundance of cyanobacteria obtained by HTS with absolute quantitative data from microscopy analysis (or from other techniques like quantitative Polymerase Chain Reaction) will be necessary to confirm *Lyngbya robusta* as the most abundant species in this lake.

In addition, more investigations will be required to elucidate the relationships between *Lyngbya robusta* and cyanobacterial-associated or free-living communities of heterotrophic bacteria in this lake, including the possible contribution of the observed modulations in microbial functional profiles to the *Lyngbya robusta* bloom. In order to better understand the role of heterotrophic bacteria in the dynamics of cyanobacterial blooms, several studies have adopted a co-culture system, which however is not fully representative of the natural environment [7, 8, 97, 98]. The co-occurrence of heterotrophic bacteria and prokaryotic phytoplankton at species level is difficult to assess and compare to other studies since it is well known that only a relatively small amount of 16S or shotgun data at the species level can be taxonomically classified. For what concerns phytoplankton, comparison of shotgun reads (using Kraken2) with the Genbank plant section, diatom genomes and green algae genomes, allowed only a very low fraction of reads (< 0.1%) to be classified as phytoplankton. However, we cannot a priori exclude its presence because the analysis might be biassed by a still very sparse level of genome sequence information concerning these organisms. Samples for a complementary microscopy analysis are unfortunately not available but might have indicated how deep the shotgun approach can provide a snapshot of the complete community, with presently available genome sequence information. For bacteria, co-occurrence network analysis (Online resource 1: Supplemental Fig. S6) of the 2017 Epilimnion genus level data (genera with ≥ 1% relative abundance) indicates a single positive interaction between *Lyngbya* and *Limnohabitans* and both positive and negative interactions between *Microcystis* and a number of poorly characterized genera. At 2.5 × Secchi depth (genera with ≥ 1% relative abundance), the interaction network is more complex with both *Lyngbya* and *Microcystis* involved in numerous positive and negative interactions (Online resource 1: Supplemental Fig. S6). Evidence of heterotrophic bacteria degrading microcystin are reported by Christoffersen et al. [99] without presenting analysis of phylogeny, while their role in the production and degradation of *Microcystis* cyanopeptides is reported by Briand et al. [100] including a phylum microbial classification. At the genus level, some heterotrophic bacteria present with low (< 1%) relative abundance in our analysis have been shown to enhance (e.g. *Flavobacterium* and *Pseudomonas*) but also inhibit (e.g. *Flavobacterium*) *Microcystis* growth while *Pseudomonas* has been described to have a cyanolytic capacity [101]. While our results have revealed a metagenome with a highly complex composition, including significant amounts of phages, they also show that a large portion of the metagenome still remains unclassified, even at higher taxonomic ranks, due to the presently still limited number of complete (or nearly complete) bacterial genomes and genome assemblies. From these results, it appears that also phages, with a rapidly growing number of known genomes [102], should be included in standard shotgun analysis methods such as Kraken2. We also identified for the first time a specific functional profile at M depth and, less frequently, in S samples suggesting that distinct metabolic processes characterize the bacterial community along the water column in Lake Varese. In particular, we observed that, among the modulated pathways, sulphur and nitrogen cycles are active processes in this lake, particularly in the M zone, characterized by poor or null oxygen content. In conclusion, the genetic and functional profile of the microbial community together with other components of the microbiome, and data on water nutrients and pollutants content could provide a more solid ground to understand the bloom dynamics, and later on, to contribute to the modelling studies for earlier prediction.

## Supplementary Information

Below is the link to the electronic supplementary material.

Supplementary file1 (PDF 3156 KB)

Supplementary file2 (XLSX 162 KB)

## Data Availability

The datasets generated during the current study are available as raw FASTQ files in the NCBI SRA archive under project ID PRJNA694367 (Shotgun data: https://www.ncbi.nlm.nih.gov/bioproject/PRJNA694367) and PRJNA694444 (16S amplicon data: https://www.ncbi.nlm.nih.gov/bioproject/PRJNA694444).
