# Supplementary material for: Metagenomics Analysis to Investigate the Microbial Communities and Their Functional Profile During Cyanobacterial Blooms in Lake Varese
Source: Microb Ecol. 2021 Nov 12;83(4):850–68. doi: 10.1007/s00248-021-01914-5 (PMC9016052; doi:10.1007/s00248-021-01914-5)
Supplement: Supplementary file 1 — Supplementary file1 (PDF 3156 KB) [file 248_2021_1914_MOESM1_ESM.pdf]

Microbial Ecology

Supplementary Information

Metagenomics analysis to investigate the microbial communities and their functional profile during Cyanobacterial Blooms in Lake Varese

Isabella Sanseverino[1], Patrizia Pretto[2], Diana Conduto António[1], Armin Lahm[3], Chiara Facca[4], Robert Loos[1], Helle Skejo[1], Andrea Beghi[5], Franca Pandolfi[5], Pietro Genoni[5], and Teresa Lettieri[1*]

[1]European Commission, Joint Research Centre (JRC), Via E. Fermi 2749, 21027 Ispra (VA), Italy

[2]Biosearch Ambiente srl, Via Tetti Gai 59, 10091, Alpignano (TO), Italy

[3]Bioinformatics project support, P.za S.M. Liberatrice 18, 00153 Roma, Italy

[4]Department of Environmental Science, Informatics and Statistics, University Ca' Foscari Venezia, Via Torino 155, 301702 Mestre (VE), Italy

[5]ARPA, Agenzia Regionale per la Protezione dell'Ambiente della Lombardia, Via Ippolito Rosellini 17, 20124, Milano, Italy

*** Correspondence:**
Teresa Lettieri
teresa.lettieri@ec.europa.eu

Supplementary Figures and Tables

Table S1. Overview of shotgun data processing. Number of shotgun data read pairs that passed the trimmomatic QC filtering step. Epi 0.5 m - E; 2.5x Secchi - S and Meso 13 m – M.

Dataset	Total # read pairs	After QC filtering
31/8/2016 E	9116300	5831783
31/8/2016 M	8539736	6744986
7/9/2016 E	8642971	6755860
7/9/2016 S	8115974	6418729
14/9/2016 E	8073606	6434912
14/9/2016 S	8354250	6606222
21/9/2016 E	10100795	7902173
21/9/2016 S	10010228	7962451
19/7/2017 E	13481272	11645493
19/7/2017 M	12130593	10550605
19/7/2017 S	10644704	9052179
26/7/2017 E	10962868	9257637
2/8/2017 E	14712626	12869176
2/8/2017 M	13299405	11306753
9/8/2017 E	11466233	9573140
9/8/2017 M	13092881	11347673
23/8/2017 E	10728850	9333322
23/8/2017 S	10588789	8997837
30/8/2017 E	12667744	10983341
30/8/2017 S	11402052	9777820
27/9/2017 E	13143807	11505318
27/9/2017 S	14233363	12319660
04/10/2017 E	11120130	9365988
04/10/2017 S	16960932	14427991

Table S2. Overview of 16S V3-V4 data processing. Number of V3-V4 amplicon sequences (merged read pairs) are reported together with minimum, average, median and maximum length of sequences. The number sequences shorter than 350 nucleotides is also reported. Epi 0.5 m - E; 2.5x Secchi - S and Meso 13 m – M.

Dataset	Min	Average	Median	Max	Number of amplicons	below 350
14/9/2016 E	383	410.0	405	440	31709	6
14/9/2016 M	367	418.8	426	465	47444	24
14/9/2016 S	351	414.3	407	464	57488	16
21/9/2016 E	373	410.6	405	464	26185	6
21/9/2016 M	366	417.5	422	443	41994	18
21/9/2016 S	371	411.8	407	463	36515	12
28/9/2016 E	360	411.3	407	457	58522	14
28/9/2016 M	353	416.8	422	463	32481	15
28/9/2016 S	371	413.7	407	442	22256	8
31/8/2016 E	372	410.4	407	441	43676	24
31/8/2016 M	373	420.0	426	465	24584	8
5/10/2016 E	358	412.4	407	465	43197	25
5/10/2016 M	353	417.5	422	463	30257	12
7/9/2016 E	373	411.1	407	464	34290	12
7/9/2016 M	353	419.5	426	465	29238	5
7/9/2016 S	368	412.2	405	465	70221	15
19/7/2017 E	351	411.5	407	450	60514	68
19/7/2017 S	355	411.5	407	451	60013	72
26/7/2017 E	372	410.9	406	472	50194	56
2/8/2017 E	371	411.4	407	478	61401	104
2/8/2017 M	356	416.7	419	464	57292	78
2/8/2017 S	353	412.3	407	443	38997	43
9/8/2017 E	354	410.9	406	467	62980	81
9/8/2017 S	363	418.7	423	474	63583	106
23/8/2017 E	366	410.7	406	474	62511	87
23/8/2017 S	371	411.8	407	455	51774	50
30/8/2017 E	368	410.2	405	462	43059	60
30/8/2017 S	358	411.0	407	479	55959	58
27/9/2017 E	372	411.0	407	459	26173	21
27/9/2017 S	356	416.6	422	464	38156	49
4/10/2017 E	357	413.3	420	439	47124	64
4/10/2017 S	351	414.1	419	480	58204	98

Table S3. Physico-chemical parameters measured in Lake Varese in 2016. Physico-chemical parameters measured at different depths (Epi 0.5 m - E; 2.5x Secchi - S and Meso 13 m - M) in Lake Varese during the indicated sampling dates. The table shows mean and standard deviation (n=3) calculated for each parameter.

**Date of Sampling**	**Parameters and Unit of Measurement**		**E (0.5 m)**	**S (*)**	**M (13 m)**
31/8/2016	Water Temperature (°C)	(WT)	25 ± 0.0	9.3 ± 0.1	9.3 ± 0.1
	Conductivity (µS/cm at 25°C)	(CD)	242 ± 0.0	313.3 ± 1.2	313.3 ± 1.2
	Dissolved oxygen (mg/L)	(DO)	9.7 ± 0.0	0.0 ± 0.0	0.0 ± 0.0
	Oxygen saturation (%)	(OS)	120.7 ± 0.4	0.0 ± 0.0	0.0 ± 0.0
	Photosynthetically active radiation (µE/m²/s)	(PAR)	126.8 ± 38.9	6 ± 0.0	6 ± 0.0
	Oxidation Reduction Potential (mV)	(ORP)	134.3 ± 0.5	-141.7 ± 1.2	-141.7 ± 1.2
	pH	pH	8.5 ± 0.0	7.1 ± 0.0	7.1 ± 0.0
7/9/2016	Water Temperature (°C)	(WT)	24.8 ± 0.0	15 ± 0.2	10.2 ± 0.1
	Conductivity (µS/cm at 25°C)	(CD)	242 ± 0.0	307.7 ± 0.6	316.3 ± 0.6
	Dissolved oxygen (mg/L)	(DO)	8.2 ± 0.0	1.1 ± 0.0	0 ± 0.0
	Oxygen saturation (%)	(OS)	101.8 ± 0.1	10.8 ± 0.0	0 ± 0
	Photosynthetically active radiation (µE/m²/s)	(PAR)	485.5 ± 275.5	13 ± 0.0	6 ± 0.0
	Oxidation Reduction Potential (mV)	(ORP)	182.5 ± 0.5	193 ± 0.0	-137.3 ± 2.3
	pH	pH	8.5 ± 0.0	7.5 ± 0.0	7.2 ± 0.0
14/9/2016	Water Temperature (°C)	(WT)	25.2 ± 0.0	16.8 ± 0.0	9.7 ± 0.0
	Conductivity (µS/cm at 25°C)	(CD)	243 ± 0.0	308.7 ± 0.6	322.3 ± 0.6
	Dissolved oxygen (mg/L)	(DO)	8.8 ± 0.0	0.1 ± 0.0	0 ± 0.0
	Oxygen saturation (%)	(OS)	109.2 ± 0.0	1.3 ± 0.1	0 ± 0.0
	Photosynthetically active radiation (µE/m²/s)	(PAR)	485.7 ± 2.5	10 ± 0.0	6 ± 0.0
	Oxidation Reduction Potential (mV)	(ORP)	192.3 ± 0.6	212 ± 0.0	-157 ± 0.0
	pH	pH	8.4 ± 0.0	7.3 ± 0.0	7 ± 0.0
21/9/2016	Water Temperature (°C)	(WT)	22.2 ± 0.0	15.8 ± 0.0	10.1 ± 0.0
	Conductivity (µS/cm at 25°C)	(CD)	248 ± 0.0	313 ± 1.0	321 ± 0.0
	Dissolved oxygen (mg/L)	(DO)	8.5 ± 0.0	0 ± 0.0	0 ± 0.0
	Oxygen saturation (%)	(OS)	100.4 ± 0.0	0.2 ± 0.1	0 ± 0.0

	Photosynthetically active radiation (μE/m²/s)	(PAR)	76.3 ± 2.5	7 ± 0.0	6 ± 0.0
	Oxidation Reduction Potential (mV)	(ORP)	205.7 ± 0.6	150.7 ± 2.9	-153.3 ± 0.6
	pH	pH	8.3 ± 0.0	7.3 ± 0.0	7.1 ± 0.0
28/9/2016	Water Temperature (°C)	(WT)	21.7 ± 0.0	12.5 ± 0.0	9.7 ± 0.0
	Conductivity (μS/cm at 25°C)	(CD)	246 ± 0.0	315 ± 0.0	318 ± 0.0
	Dissolved oxygen (mg/L)	(DO)	9 ± 0.0	0 ± 0.0	0 ± 0.0
	Oxygen saturation (%)	(OS)	103.9 ± 0.1	0 ± 0.0	0 ± 0.0
	Photosynthetically active radiation (μE/m²/s)	(PAR)	195 ± 18.4	6 ± 0.0	6 ± 0.0
	Oxidation Reduction Potential (mV)	(ORP)	155 ± 0.0	-14.3 ± 9.2	-175 ± 0.0
	pH	pH	8.5 ± 0.0	7.2 ± 0.0	7.1 ± 0.0
5/10/2016	Water Temperature (°C)	(WT)	20.4 ± 0.0	10 ± 0.0	10 ± 0.0
	Conductivity (μS/cm at 25°C)	(CD)	252 ± 0.0	319 ± 0.0	319 ± 0.0
	Dissolved oxygen (mg/L)	(DO)	8 ± 0.0	0 ± 0.0	0 ± 0.0
	Oxygen saturation (%)	(OS)	91.3 ± 0.1	0 ± 0.0	0 ± 0.0
	Photosynthetically active radiation (μE/m²/s)	(PAR)	2265.3 ± 154.7	6 ± 0.0	6 ± 0.0
	Oxidation Reduction Potential (mV)	(ORP)	-51.3 ± 0.6	-152.0 ± 2	-152.0 ± 2
	pH	pH	8.3 ± 0.0	7.2 ± 0.0	7.2 ± 0.0

(*) S depth: 13 m on 31/8/2016; 7.6 m on 7/9/2016; 8 m on 14/9/2016; 7.8 m on 21/9/2016; 10.3 m on 28/9/2016 and 13 m on 5/10/2016

Table S4. Physico-chemical parameters measured in Lake Varese in 2017. Physico-chemical parameters measured at different depths (Epi 0.5 m - E; 2.5x Secchi - S and Meso 13 m - M) in Lake Varese during the indicated sampling dates. The table shows mean and standard deviation (n=3) of each sampling campaign, except for the campaign on 2/8/2017 where only one measurement was taken at each depth.

**Date of Sampling**	**Parameter and Unit of Measurements**		**E (0.5 m)**	**S (*)**	**M (13 m)**
2/8/2017	Water Temperature (°C)	(WT)	27.2	26.4	9.5
	Conductivity (µS/cm at 25°C)	(CD)	n.d.	210.4	224.2
	Dissolved oxygen (mg/L)	(DO)	10.54	11.2	0.5
	Oxygen saturation (%)	(OS)	133	139.5	4.5
	Photosynthetically active radiation (µE/m²/s)	(PAR)	n.d.	235.9	0
	Oxidation Reduction Potential (mV)	(ORP)	n.d.	26.4	-125.7
	pH	pH	8.9	8.9	7.5
9/8/2017	Water Temperature (°C)	(WT)	25.6 ± 0.0	24 ± 0.0	8.7 ± 0.0
	Conductivity (µS/cm at 25°C)	(CD)	205.3 ± 0,6	236.7 ± 1,2	306 ± 0,0
	Dissolved oxygen (mg/L)	(DO)	8.3 ± 0,0	5 ± 0.1	0.27 ± 0.0
	Oxygen saturation (%)	(OS)	102.7 ± 0.2	60 ± 0.8	2.3 ± 0.0
	Photosynthetically active radiation (µE/m²/s)	(PAR)	442.3 ± 1.2	9 ± 0.0	6 ± 0.0
	Oxidation Reduction Potential (mV)	(ORP)	229.7 ± 0.6	217 ± 0.0	-164.3 ± 0.6
	pH	pH	8.7 ± 0.0	7.9 ± 0.0	7.2 ± 0.0
23/8/2017	Water Temperature (°C)	(WT)	25.1 ± 0.0	24.3 ± 0.0	9.2 ± 0.0
	Conductivity (µS/cm at 25°C)	(CD)	195 ± 0.0	208.7 ± 0.6	306 ± 0.0
	Dissolved oxygen (mg/L)	(DO)	11.1 ± 0.0	9.2 ± 0.2	0.3 ± 0.0
	Oxygen saturation (%)	(OS)	136.3 ± 0,2	111 ± 2.7	2.3 ± 0.0
	Photosynthetically active radiation (µE/m²/s)	(PAR)	155.3 ± 15.6	21 ± 0.0	6 ± 0.0
	Oxidation Reduction Potential (mV)	(ORP)	283.3 ± 0.6	262 ± 0.0	-157 ± 0.0
	pH	pH	9.1 ± 0.0	8.5 ± 0.0	7.2 ± 0.0
30/8/2017	Water Temperature (°C)	(WT)	26.1 ± 0.0	23.9 ± 0.0	9.2 ± 0.0
	Conductivity (µS/cm at 25°C)	(CD)	172.7 ± 0.6	239.7 ± 0.6	312 ± 0.0
	Dissolved oxygen (mg/L)	(DO)	8.8 ± 0.1	2.2 ± 0.4	0.2 ± 0.0

	Oxygen saturation (%)	(OS)	110 ± 1.2	26.3 ± 5.1	1.7 ± 0.0
	Photosynthetically active radiation (μE/m²/s)	(PAR)	225.7 ± 6.4	12 ± 0.0	6 ± 0.0
	Oxidation Reduction Potential (mV)	(ORP)	249.3 ± 1.2	273 ± 0.0	-39.7 ± 0.6
	pH	pH	7.7 ± 0.0	6.3 ± 0.0	5.6 ± 0.0
27/9/2017	Water Temperature (°C)	(WT)	19.9 ± 0.0	15.3 ± 0.1	9.8 ± 0.0
	Conductivity (μS/cm at 25°C)	(CD)	185 ± 0.0	273 ± 0.0	281.3 ± 0.6
	Dissolved oxygen (mg/L)	(DO)	10.2 ± 0.0	0.5 ± 0.0	0.3 ± 0.0
	Oxygen saturation (%)	(OS)	113.9 ± 0.2	5.1 ± 0.1	2.4 ± 0.0
	Photosynthetically active radiation (μE/m²/s)	(PAR)	151.7 ± 2.3	8 ± 0.0	6 ± 0.0
	Oxidation Reduction Potential (mV)	(ORP)	134 ± 0.0	170.3 ± 0.6	-163.7 ± 1.2
	pH	pH	8.6 ± 0.0	7.3 ± 0.0	7.2 ± 0.0
4/10/2017	Water Temperature (°C)	(WT)	19.5 ± 0.0	10.8 ± 0.0	9.5 ± 0.0
	Conductivity (μS/cm at 25°C)	(CD)	186 ± 0.0	281 ± 0.0	284 ± 0.0
	Dissolved oxygen (mg/L)	(DO)	10.5 ± 0.0	0.3 ± 0.0	0.3 ± 0.0
	Oxygen saturation (%)	(OS)	115.9 ± 0.1	2.4 ± 0.0	2.4 ± 0.0
	Photosynthetically active radiation (μE/m²/s)	(PAR)	388 ± 0.0	6 ± 0.0	6 ± 0.0
	Oxidation Reduction Potential (mV)	(ORP)	128 ± 0.0	-10.3 ± 0.6	-48.3 ± 0.6
	pH	pH	8.9 ± 0.0	7.3 ± 0.0	7.2 ± 0.0

(*) S depth corresponded to the water depth of 2 m on 2/8/2017; 6 m on 9/8/2017; 5 m on 23/8/2017 and 30/8/2017; 8.75 m on 27/9/2017 and 11 m on 4/10/2017. n.d. stands for not determined

Table S5. Nutrients measured in Lake Varese. Nutrients measured at different depths (Epi 0.5 m - E; 2.5x Secchi - S and Meso 13 m - M) in Lake Varese during the indicated sampling dates.

**Date of Sampling and Depth of Measurements**		**Nutrients (mg/L)**			
	Laboratory	**NO_3^-**	**NH_4^+**	**SO_4^{2-}**	**Total Phosphorus**
	LOD (BO)	n.d.	n.d.	n.d.	0.05
	LOD (JRC)	0.049	0.02	0.002	n.d.
	LOQ (JRC)	0.086	0.02	0.004	n.d.
31/8/2016 E	BO	n.d.	n.d.	n.d.	< LOD
31/8/2016 M	JRC	< LOD	0.468	7.1	n.d.
31/8/2016 S	JRC	< LOD	0.468	7.1	n.d.
7/9/2016 E	BO	n.d.	n.d.	n.d.	0.08
7/9/2016 S	JRC	< LOD	< LOD	6.9	n.d.
7/9/2016 M	JRC	< LOD	0.391	6.9	n.d.
14/9/2016 E	BO	n.d.	n.d.	n.d.	0.06
14/9/2016 M	JRC	< LOD	0.51	6.4	n.d.
21/9/2016 E	BO	n.d.	n.d.	n.d.	0.06
21/9/2016 S	JRC	0.062	< LOD	6.6	n.d.
21/9/2016 M	JRC	< LOD	0.417	6.5	n.d.
28/9/2016 E	BO	n.d.	n.d.	n.d.	0.26
5/10/2016 E	BO	n.d.	n.d.	n.d.	< LOD
19/7/2017 E	JRC	< LOD	< LOD	6.7	n.d.
19/7/2017 S	JRC	< LOD	< LOD	6.8	n.d.
19/7/2017 M	JRC	0.066	0.311	6.8	n.d.
26/7/2017 E	JRC	< LOD	< LOD	5.5	n.d.
26/7/2017 S	JRC	< LOD	< LOD	6.5	n.d.
26/7/2017 M	JRC	< LOD	0.319	7	n.d.
2/8/2017 E	JRC	< LOD	< LOD	6.5	n.d.
2/8/2017 S	JRC	< LOD	< LOD	6.3	n.d.
2/8/2017 M	JRC	< LOD	< LOD	6.3	n.d.
9/8/2017 E	JRC	< LOD	< LOD	6.1	n.d.
9/8/2017 S	JRC	< LOD	< LOD	6.2	n.d.
9/8/2017 M	JRC	< LOD	0.545	6	n.d.
23/8/2017 E	JRC	< LOD	< LOD	6	n.d.
23/8/2017 S	JRC	< LOD	< LOD	6	n.d.
23/8/2017 M	JRC	< LOD	0.47	5.3	n.d.
30/8/2017 E	JRC	< LOD	< LOD	5.7	n.d.
30/8/2017 S	JRC	< LOD	< LOD	5.9	n.d.

30/8/2017 M	JRC	< LOD	0.65	4.6	n.d.
27/9/2017 E	JRC	< LOD	< LOD	5.5	n.d.
27/9/2017 S	JRC	< LOD	0.07	5.8	n.d.
27/9/2017 M	JRC	< LOD	0.676	2.3	n.d.
4/10/2017 E	JRC	< LOD	< LOD	5.7	n.d.
4/10/2017 S	JRC	< LOD	0.406	3.3	n.d.
4/10/2017 M	JRC	< LOD	0.831	2.1	n.d.

(*) S depth: 13 m on 31/8/2016; 7.6 m on 7/9/2016; 7.8 m on 21/9/2016; 5m on 19/7/2017 and 26/7/2017; 2 m on 2/8/2017; 6 m on 9/8/2017; 5 m on 23/8/2017 and 30/8/2017; 8.75 m on 27/9/2017 and 11 m on 4/10/2017. n.d. stands for not determined. LOD stands for Limit of Detection, LOQ stands for Limit of Quantification.

Table S6. Chlorophyll *a* content measured in Lake Varese in 2016. Chlorophyll *a* (Chl*a*) content measured at different depths (Epi 0.5 m - E; 2.5x Secchi - S and Meso 13 m - M) in Lake Varese during the indicated sampling dates.

	Chl*a* content (µg/L)		
Date of sampling	**E**	**S (*)**	**M**
**31/8/2016**	6.65	1.60	1.60
**7/9/2016**	5.10	14.52	2.04
**14/9/2016**	5.17	15.42	4.72
**21/9/2016**	12.84	10.23	4.05
**28/9/2016**	1.48	3.80	0.81
**5/10/2016**	3.35	4.15	4.15
**Average (Avg)**	5.77	8.29	2.89

(*) S depth: 13 m on 31/8/2016; 7.6 m on 7/9/2016; 8 m on 14/9/2016; 7.8 m on 21/9/2016; 10.3 m on 28/9/2016 and 13 m on 5/10/2016

Table S7. Chlorophyll *a* content measured in Lake Varese in 2017. Chlorophyll *a* (Chl*a*) content measured at different depths (Epi 0.5 m - E; 2.5x Secchi - S and Meso 13 m - M) in Lake Varese during the 2017 campaign.

	Chl*a* content (µg/L)		
Date of sampling	**E**	**S (*)**	**M**
**19/7/2017**	10.70	7.10	2.90
**26/7/2017**	18.30	18.14	3.30
**2/8/2017**	7.00	14.00	22.00
**9/8/2017**	19.20	13.20	2.40
**23/8/2017**	12.00	19.00	2.80
**30/8/2017**	14.20	22.40	3.20
**27/9/2017**	11.00	7.20	7.40
**4/10/2017**	4.10	7.90	3.00
**Average (Avg)**	12.06	13.62	5.88

(*) S depth: 5 m on 19/7/2017, 26/7/2017; 2 m on 2/8/2017; 6 m on 9/8/2017; 5 m on 23/8/2017 and 30/8/2017; 8.75 m on 27/9/2017 and 11 m on 4/10/2017

Table S8. MegaHit shotgun contig overview. Number, length and N50 value of MegaHit contigs obtained from the shotgun data. Also reported is the number of contigs in the indicated length interval. Epi 0.5 m - E; 2.5x Secchi - S and Meso 13 m – M.

			Contig length					
**Dataset**	**# of contigs**	**max length**	**> 100000**	**> 50000**	**> 10000**	**>5000**	**>=1000**	**<1000**
31/8/2016 E	73519	42318			33	90	5208	68188
31/8/2016 M	76356	51444		1	322	690	7252	68091
7/9/2016 E	76356	51444		1	322	690	7252	68091
7/9/2016 S	76760	67674		2	52	248	8126	68332
14/9/2016 E	80943	67784		2	92	451	9865	70533
14/9/2016 S	63170	136338	3	9	113	155	5105	57785
21/9/2016 E	97602	377841	13	19	147	248	8916	88259
21/9/2016 S	125014	83200		1	192	482	14299	110040
19/7/2017 E	446482	152833	7	77	1213	2552	58178	384455
19/7/2017 M	460474	286300	16	53	1249	2634	60581	395941
19/7/2017 S	345883	189922	5	34	952	2060	43741	299091
26/7/2017 E	367568	183641	5	39	953	2045	46146	318380
2/8/2017 E	475171	204199	6	51	1391	2899	57266	413558
2/8/2017 M	426540	222241	26	76	867	2181	53888	369502
9/8/2017 E	358842	204210	6	60	966	2021	48052	307737
9/8/2017 M	443524	394439	18	53	1233	3105	56841	382274
23/8/2017 E	332128	217027	24	59	1161	2436	43211	285237
23/8/2017 S	310075	224316	18	73	1054	2072	42605	264253
30/8/2017 E	361139	220278	12	65	982	2013	43278	314789
30/8/2017 S	370783	314525	11	73	1049	2104	45455	322091
27/9/2017 E	431948	249947	14	52	1217	2601	60031	368033
27/9/2017 S	459518	154350	5	24	1351	3233	56569	398336
4/10/2017 E	362357	282165	12	28	952	2600	50995	307770
4/10/2017 S	445017	821984	28	64	943	2248	51512	390222

Table S9. Conservation of MegaHit shotgun contigs in samples. Conservation of MegaHit contigs across all samples collected in 2017 as determined by BLASTN. Only matches with 99% sequence identity and with a match alignment length ≥ 50% of the length of the query contig are reported. Values are contig coverage values (displayed as rounded integers) obtained from MegaHit for the matched sample normalized for the total number of read pairs in each sample. Rightmost column: Lr = *Lyngbya robusta* genome assembly (*Limnoraphis robusta CS-951.)*; L. sp = *Lyngbya* spp. *PCC 8106 1099428180450).* Only a subset of matching contigs (longest) are shown due to space limitations. Sample 30/8/2017 E with the highest number of *Lyngbya robusta* matches in the Kraken2 analysis is indicated in yellow. Epi 0.5 m - E; 2.5x Secchi - S and Meso 13 m – M.

Contig	Length	19/7/2017 E	19/7/2017 S	19/7/2017 M	26/7/2017 E	2/8/2017 E	2/8/2017 M	9/9/2017 E	9/8/2017 M	23/8/2017 E	23/8/2017 S	30/8/2017 E	30/8/2017 S	27/9/2017 E	27/9/2017 S	4/10/2017 E	4/10/2017 S	Genome
k141_237689_9/8/2017 E	137961	6			10			9		6	9	12	8	11				Lr
k141_920_26/7/2017 E	132558	6			10			9						11				Lr
k141_80290_23/8/2017 S	130786										9							Lr
k141_56948_23/8/2017 E	129595							9		6	9	12	8	11				Lr
k141_276541_19/7/2017 E	118925	7			11					6	9	12		10				Lr
k141_127895_19/7/2017 E	115593	6			10		5	9		6	9	12	8	11				Lr
k141_187606_9/8/2017 E	108071						5	9		6	9	12	8	9				Lr
k141_29433_23/8/2017 E	107952						5	9		6	9	12	8					Lr
k141_182921_30/8/2017 E	98087							9			9	12	8					Lr
k141_183280_30/8/2017 E	97073							9		6	9	12	8					Lr
k141_62685_27/9/2017 E	95824	6			10		5	9		6	9	12	8	11				Lr
k141_4749_2/8/2017 M	92338						5	9		6	9	12	8	9				Lr
k141_26964_30/8/2017 E	91750						5	9		6	9	12	8					Lr
k141_64076_27/9/2017 E	91715						5	9						9				Lr
k141_3669_23/8/2017 E	91333							9		6	9	12	8					Lr
k141_29439_19/7/2017 E	91304	7																Lr
k141_149995_9/8/2017 E	91026							9		6	9	12	8					Lr
k141_45444_19/7/2017 E	87494	7					5							9				Lr
k141_15288_23/8/2017 S	85075										9							Lr
k141_187559_30/8/2017 E	84966									7		12						Lr
k141_55121_23/8/2017 S	84824	6			10		5	9		6	9	12	8	11				Lr
k141_92268_30/8/2017 S	84794	6			10		5	9		6	9	12	8	11				Lr
k141_64012_30/8/2017 E	84528	6			10		5	9		6	9	12	8	11				Lr
k141_4042_30/8/2017 S	84237				11			9		6	9	12	8	10				Lr
k141_39993_9/8/2017 E	83609	7			11			9		6	9	12	8	10				Lr
k141_272525_27/9/2017 E	83419	7	5		10			9		6	9	12	9	9				Lr
k141_167885_23/8/2017 S	83411	7			11			9		6	9	12	8	10				Lr
k141_102025_30/8/2017 E	83411	7			11			9		6	9	12	8	10				Lr
k141_219532_26/7/2017 E	83392	7			11			9		6	9	12	8	10				Lr
k141_145744_23/8/2017 E	83195						5	9		7	9	12	8	9				Lr
k141_230547_27/9/2017 E	83083	7			11			9		6	9	12	8	10				Lr
k141_15786_23/8/2017 E	82525	7			11			9		6	9	12	8	10				Lr
k141_7683_30/8/2017 E	82274						5	9		7	9	12	8	9				Lr
k141_115161_23/8/2017 S	82227						5	9		7	9	12	8	9				Lr
k141_124844_30/8/2017 S	81537						5	9		7	9	12	8	9				Lr
k141_41843_30/8/2017 S	81053	6								6	9	12	8					Lr
k141_153488_27/9/2017 E	79271						5			7	9	12	8	9				Lr
k141_143859_19/7/2017 E	77837	7																Lr
k141_59614_30/8/2017 E	77625											12						Lr
k141_211040_30/8/2017 E	77440				11		5	9		7	9	12	8	10				Lr
k141_185490_23/8/2017 S	77440				11		5	9		7	9	12	8	10				Lr
k141_220184_30/8/2017 S	77440				11		5	9		7	9	12	8	10				Lr
k141_62850_9/8/2017 E	77337				11		5	9		7	9	12	8	10				Lr
k141_52389_23/8/2017 S	77212										9		8	10				Lr
k141_157016_30/8/2017 E	76892						5			7	9	12	8	9				Lr
k141_50119_23/8/2017 S	76860						5			7	9	12	8	9				Lr
k141_188362_30/8/2017 S	75820	7						9		6	9	12	8					Lr
k141_810_9/8/2017 E	75730	7						9		6	9	12	8					Lr
k141_68736_30/8/2017 E	75646	7						9		6	9	12	8					Lr
k141_28556_23/8/2017 S	75402						5	9		6	9	12	8	9				Lr

Contig	Length	19/7/2017 E	19/7/2017 S	19/7/2017 M	26/7/2017 E	2/8/2017 E	2/8/2017 M	9/8/2017 E	9/8/2017 M	23/8/2017 E	23/8/2017 S	30/8/2017 E	30/8/2017 S	27/9/2017 E	27/9/2017 S	4/10/2017 E	4/10/2017 S	Genome
k141_88875_23/8/2017 E	75398	7						8		6	9	12	8					Lr
k141_3751_30/8/2017 S	75242						5	8		6	9	12	8	9				Lr
k141_151132_23/8/2017 E	74738				11		5	8		7	10	12	8	10				Lr
k141_113406_27/9/2017 E	74707	7								6		11	8	9				Lr
k141_19724_19/7/2017 E	74007	7	5		10			9		6	10	12	9	9				Lr
k141_158256_19/7/2017 E	72755	7						8										Lr
k141_2354_23/8/2017 S	72737							8		6	9	12	8					Lr
k141_5103_30/8/2017 E	72541									6	9	12	8					Lr
k141_153053_30/8/2017 S	72448	7	5		10		5	9		6	10	12	9	9				Lr
k141_29465_19/7/2017 E	72429	7									9			9				Lr
k141_112782_23/8/2017 S	72298	7						9			9	12	8	9				Lr
k141_128648_30/8/2017 E	72298							9			9	12	8	9				Lr
k141_125401_30/8/2017 S	72298							9			9	12	8	9				Lr
k141_16852_23/8/2017 S	72246	7			10	4	6	9		6	10	12	8	10				Lr
k141_198644_23/8/2017 S	71633	7			11		5	9		6	9	12	8	9				Lr
k141_14255_2/8/2017 M	71590	7			11		5	9		6	9	12	8	9				Lr
k141_170572_30/8/2017 E	71532	7	5		10		5	9		6	10	12	9	9				Lr
k141_56426_23/8/2017 E	71521	7	5		10		5	9		6	10	12	9	9				Lr
k141_17631_9/8/2017 E	71478	7	5		10		5	9		6	10	12	9	9				Lr
k141_201860_23/8/2017 S	71452	7	5		10		5	9		6	10	12	9	9				Lr
k141_290682_27/9/2017 E	70672	7			11		5	9		6	9	12	8	9				Lr
k141_294067_19/7/2017 E	70672	7			11		5	9		6	9	12	8	9				Lr
k141_138053_30/8/2017 E	68679	7			11		5	9		6	9	12	8	9				Lr
k141_130544_23/8/2017 E	68679	7			11		5	9		6	9	12	8	9				Lr
k141_146227_30/8/2017 S	68679	7			11		5	9		6	9	12	8	9				Lr
k141_159774_26/7/2017 E	68540	7			11		5	9		6	9	12	8	9				Lr
k141_38246_9/8/2017 E	68540	7			11		5	9		6	9	12	8	9				Lr
k141_87536_9/8/2017 E	68158							9			9	12	8	9				Lr
k141_174263_23/8/2017 S	68086	6						9		6	9	12	8					Lr
k141_23254_2/8/2017 M	68063	7					5							9				Lr
k141_8330_9/8/2017 E	66921	7						8		6	10	12	8	9				Lr
k141_71337_30/8/2017 S	66677	7						8		6	10	12	8	9				Lr
k141_92856_27/9/2017 E	66677	7						8		6	10	12	8	9				Lr
k141_73849_30/8/2017 E	66677	7						8		6	10	12	8	9				Lr
k141_22592_27/9/2017 E	66664	7			11		5	9		6	9	13	8	9				Lr
k141_267174_2/8/2017 M	65736						5	8		7	10	12	8	8				Lr
k141_244830_27/9/2017 E	64167	6			10			8		6	9	12	8	9				Lr
k141_181443_23/8/2017 S	62849	7			10		5	9		7	9	12	8	9				Lr
k141_89388_23/8/2017 S	62472	7					6	8		6	10	12	8	9				Lr
k141_230676_30/8/2017 E	62401						6			6		11						Lr
k141_196038_23/8/2017 E	62383				10			8		6	9	12	8	9				Lr
k141_49498_19/7/2017 E	62336	6			10		6											Lr
k141_14982_9/8/2017 E	61075				10		5	8		7	9	12	8	9				Lr
k141_160653_19/7/2017 E	60384	7			10	3	5	9		7	10	12	8	9				Lr
k141_126538_27/9/2017 E	60384	7			10	3	5	9		7	10	12	8	9				Lr
k141_131729_30/8/2017 S	60378	7			10	3	5	9		7	10	12	8	9				Lr
k141_214681_23/8/2017 E	60378	7			10	3	5	9		7	10	12	8	9				Lr
k141_115755_30/8/2017 E	60378	7			10	3	5	9		7	10	12	8	9				Lr
k141_154914_9/8/2017 E	60378	7			10	3	5	9		7	10	12	8	9				Lr
k141_170237_2/8/2017 M	60378	7			10	3	5	9		7	10	12	8	9				Lr

Contig	Length	19/7/2017 E	19/7/2017 S	19/7/2017 M	26/7/2017 E	2/8/2017 E	2/8/2017 M	9/8/2017 E	9/8/2017 M	23/8/2017 E	23/8/2017 S	30/8/2017 E	30/8/2017 S	27/9/2017 E	27/9/2017 S	4/10/2017 E	4/10/2017 S	Genome
k141_97764_23/8/2017 S	60378	7			10	3	5	9		7	10	12	8	9				Lr
k141_42996_27/9/2017 E	59527	7	4				5	9		7	9	12	8	9				Lr
k141_231381_27/9/2017 E	58703				10		5	8		7	9	12	8	9				Lr
k141_10028_30/8/2017 E	58502	7								6		11	8	9				Lr
k141_14864_23/8/2017 S	58426	7	5		11		5	9		6	9	13	8	9				Lr
k141_127019_9/8/2017 E	58308	7	5		11		5	9		6	9	13	8	9				Lr
k141_156895_9/8/2017 E	58298	7			10		5	8		6	9	11	8	9				Lr
k141_3023_23/8/2017 E	58269	7	5		11		5	9		6	9	13	8	9				Lr
k141_3035_30/8/2017 E	58208	7	5		11		5	9		6	9	13	8	9				Lr
k141_15581_30/8/2017 S	58169	7	5		11		5	9		6	9	13	8	9				Lr
k141_117192_27/9/2017 E	58169	7					5	9			9	12	8	9				Lr
k141_128806_26/7/2017 E	58155	7	5		11		5	9		6	9	13	8	9				Lr
k141_3739_19/7/2017 E	58145	7	5		11		5	9		6	9	13	8	9				Lr
k141_71561_30/8/2017 E	57809							9		7	9	12	8					Lr
k141_64752_23/8/2017 E	57678				10			9		7	9	12	8					Lr
k141_83188_30/8/2017 S	57601						5	9			10	12	8	9				Lr
k141_96267_30/8/2017 S	57504	7								6		11	8	9				Lr
k141_47540_9/8/2017 E	57411						5	9			10	12	8	9				Lr
k141_48211_23/8/2017 S	57411						5	9			10	12	8	9				Lr
k141_61240_30/8/2017 E	57411						5	9			10	12	8	9				Lr
k141_232947_19/7/2017 E	57316	7								6		11	8	9				Lr
k141_44458_23/8/2017 E	56615						5	8		6	9	12	8	9				Lr
k141_12450_9/8/2017 E	56580						5	8		6	9	12	8	9				Lr
k141_158652_30/8/2017 S	56566						5	8		6	9	12	8	9				Lr
k141_48231_30/8/2017 S	56335	7			11	4	5	10		6	9	12	8	10	3			Lr
k141_63896_26/7/2017 E	56172	7			11							12						Lr
k141_222918_2/8/2017 M	56164	6			11		5	9		7	9	12	8	9				Lr
k141_12696_2/8/2017 M	56125	7			10		5	8			9	11	8	9				Lr
k141_272756_19/7/2017 E	56008	7	4		10		5	8			9	11	8	9				Lr
k141_23529_2/8/2017 M	55503	7					5	8		6	9	12	8					Lr
k141_57056_30/8/2017 S	55470	7			10		5	8			9	11	8	9				Lr
k141_124328_26/7/2017 E	55252	7			10					6	9	12						Lr
k141_121798_30/8/2017 E	55243				10			9		6	9	12	8	9				Lr
k141_114044_23/8/2017 E	55227				10			9		6	9	12	8	9				Lr
k141_65569_26/7/2017 E	55223	7			10		5	8			9	11	8	9				Lr
k141_162582_19/7/2017 E	55132	7			10			9					8					Lr
k141_65133_19/7/2017 E	54776	7			10			9		6	10	12	8					Lr
k141_44064_26/7/2017 E	54776	7			10			9		6	10	12	8					Lr
k141_58288_23/8/2017 E	54770	7			10			9		6	10	12	8					Lr
k141_12273_30/8/2017 E	54770	7			10			9		6	10	12	8					Lr
k141_62331_9/8/2017 E	54770	7			10			9		6	10	12	8					Lr
k141_32309_30/8/2017 E	54756										9	12	8					Lr
k141_55354_30/8/2017 S	54701	7			10			9		6	10	12	8					Lr
k141_224974_27/9/2017 E	54618						5	8		6	9	12	8	9				Lr
k141_70521_19/7/2017 E	54570	7																Lr
k141_235448_30/8/2017 S	54499												8	9				Lr
k141_257873_2/8/2017 M	54362						6	8		6	9	11						Lr
k141_158650_27/9/2017 E	54259												8	9				Lr
k141_204596_23/8/2017 S	54077						6			6	9	11						Lr
k141_216821_9/8/2017 E	53979	7						9		6	9	12	8	9				Lr

Contig	Length	19/7/2017 E	19/7/2017 S	19/7/2017 M	26/7/2017 E	2/8/2017 E	2/8/2017 M	9/8/2017 E	9/8/2017 M	23/8/2017 E	23/8/2017 S	30/8/2017 E	30/8/2017 S	27/9/2017 E	27/9/2017 S	4/10/2017 E	4/10/2017 S	Genome
k141_120143_30/8/2017 S	53963	7						9		6	9	12	8	9				Lr
k141_201485_23/8/2017 S	53007	7			10		5	9		7	9	12	8	10				Lr
k141_130670_19/7/2017 E	52893	6			10			9		6	9	12	8					Lr
k141_172625_27/9/2017 E	52239							9		7	9	12	8	9				Lr
k141_3912_23/8/2017 S	52053							9		7	9	12	8	9				Lr
k141_35086_26/7/2017 E	51581	7	5		10		5	9		6	10	12	9	9				Lr
k141_133349_30/8/2017 S	51434							9		7	9	12	8	9				Lr
k141_184796_9/8/2017 E	51139							9		7	9	12	8	9				Lr
k141_81470_30/8/2017 S	50774							8		6	9	12	7					Lr
k141_5242_9/8/2017 E	50633							8		6	9	12	7					Lr
k141_62274_30/8/2017 E	50624							8		6	9	12	7					Lr
k141_187439_23/8/2017 S	50573	7			11	3	5	9		7	10	12	8	9				Lr
k141_70335_19/7/2017 E	50556	7			11	3					10							Lr
k141_155875_19/7/2017 E	50436	7			11	3	5	8		6	10	13	8	9	3			Lr
k141_156283_27/9/2017 E	50383	7					5	9		7	10	12	8	9				Lr
k141_16841_19/7/2017 S	50346	7	5		10		5	9		6	10	12	9	9				Lr
k141_64361_30/8/2017 S	50227									7	9	12	8	9				Lr
k141_111146_23/8/2017 S	48249				10					7	9	12	8					L.sp
k141_131042_30/8/2017 E	48249				10					7	9	12	8					L.sp
k141_143908_30/8/2017 S	48249				10					7	9	12	8					L.sp
k141_159016_23/8/2017 E	33537		4		10			8		7	9	12	8	9	3			L.sp
k141_81587_19/7/2017 E	13496	6			6									3				L.sp
k141_189837_9/8/2017 E	13298		4		10		5	9		7	9	12	8	9	3			L.sp
k141_196437_27/9/2017 E	13298		4		10		5	9		7	9	12	8	9	3			L.sp
k141_182631_26/7/2017 E	13238	6	4		10	3	5	9		7	9	12	8	9	3			L.sp
k141_214459_2/8/2017 M	13164	6	4		10	3	5	9		7	9	12	8	9	3			L.sp
k141_36540_27/9/2017 S	12550	6	4		10	3	5	9		7	9	12	8	9	3			L.sp

Table S10. GTDB genomes with high similarity to assembled contigs. Comparison of MegaHit contigs with GTDB genome/genome assembly sequences by BLASTN. Only matches with at least 90% identity and alignment length ≥10000 nucleotides were accepted. Column "Found" reports the number of MegaHit contigs matching the indicated genome/genome assembly. Maximal number of hits are observed for the *Lyngbya robusta* (*Limnoraphis robusta CS-951)* genome assembly indicated in yellow.

Assembly	Accession No	Found	Phylum	Description
GCA_001437905	LIBZ01000001.1	91	Actinobacteria	Actinobacteria bacterium BACL4 MAG-120920-bin74 isolate BACL14 MAG-120920-bin74 contig00003.0_to_4_1_120920-bin74, whole genome shotgun sequence
GCF_002287885	NZ_CP016768.2	91	Actinobacteria	Candidatus Nanopelagicus limnes isolate MMS-21-122 chromosome, complete genome
GCA_001437855	LIBD01000001.1	63	Actinobacteria	Actinobacteria bacterium BACL4 MAG-120820-bin23 contig00003.1_to_5_120820-bin23, whole genome shotgun sequence
GCA_001437755	LIBF01000001.1	29	Actinobacteria	Actinobacteria bacterium BACL4 MAG-121001-bin59 contig00001.1_to_4_121001-bin59, whole genome shotgun sequence
GCF_002284895	LIBG01000001.1	15	Actinobacteria	Actinobacteria bacterium BACL4 MAG-121022-bin9 contig00003_1_121022-bin9, whole genome shotgun sequence
GCF_002288185	NZ_CP016776.1	14	Actinobacteria	Candidatus Planktophila vernalis isolate MMS-IIA-15 chromosome, complete genome
GCA_001439225	LICQ01000001.1	13	Actinobacteria	Actinobacteria bacterium BACL4 MAG-120507-bin0 isolate BACL1 MAG-120507-bin0 contig00003.0_to_3_1_120507-bin0, whole genome shotgun sequence
GCA_000294575	KB912733.1	7	Actinobacteria	Actinobacterium SCGC AAA027-J17 genomic scaffold A027J17DRAFT_NODE-unique_10_len_38730.10, whole genome shotgun sequence
GCA_001439025	LIAT01000001.1	5	Actinobacteria	Actinobacteria bacterium BACL2 MAG-120813-bin23 contig00047_1_120813-bin23, whole genome shotgun sequence
	AJWB01000001.1	2	Actinobacteria	Actinobacterium SCGC AAA027-L06 A27L6_contig00009, whole genome shotgun sequence
GCA_000383815	NZ_CP015605.1	1	Actinobacteria	Actinobacteria bacterium IMCC19121 chromosome, complete genome
GCF_002288305	NZ_CP016779.1	1	Actinobacteria	Candidatus Nanopelagicus abundans isolate MMS-IIB-91 chromosome, complete genome
GCA_002366845	DFCA01000027.1	1	Actinobacteria	TPA_asm: Actinobacteria bacterium UBA3066 UBA3066_contig_1007, whole genome shotgun sequence
GCF_000020465	NC_010803.1	111	Bacteroidota	Chlorobium limicola DSM 245, complete genome
GCA_002293105	DBCP01000117.1	63	Bacteroidota	TPA_asm: Bacteroidetes bacterium UBA955 UBA955_contig_102032, whole genome shotgun sequence
GCF_001747405	NZ_CP017305.1	54	Bacteroidota	Chlorobaculum limnaeum strain DSM 1677, complete genome
GCA_002293045	DBCM01000162.1	18	Bacteroidota	TPA_asm: Flavobacteriales bacterium UBA958 UBA958_contig_10488, whole genome shotgun sequence
GCF_001509575	NZ_LMBR01000001.1	5	Bacteroidota	Chlorobium limicola strain Frasassi contig-124_0, whole genome shotgun sequence
GCF_000015125	NC_008639.1	1	Bacteroidota	Chlorobium phaeobacteroides DSM 266, complete genome
GCF_000972705	NZ_LATL02000001.1	538	Cyanobacteria	Limnoraphis robusta CS-951 contig001, whole genome shotgun sequence
GCF_000169095	NZ_AAVU01000110.1	10	Cyanobacteria	Lyngbya sp. PCC 8106 1099428180450, whole genome shotgun sequence
GCA_000179235	NZ_ADXL01000094.1	1	Cyanobacteria	Synechococcus sp. CB0101 contig08714, whole genome shotgun sequence
GCF_002252705	NZ_NQLA01000001.1	1	Cyanobacteria	Vulcanococcus limneticus LL NODE_10__length_226416_cov_7.02386, whole genome shotgun sequence
GCF_002688585	NZ_CP024034.1	230	Proteobacteria	Candidatus Fonsibacter ubiquis isolate LSUCC0530 chromosome, complete genome
GCF_000981505	NZ_LN827929.1	82	Proteobacteria	Candidatus Methylopumilus planktonicus genome assembly Candidatus Methylopumilus planktonicus MMS-2-53, chromosome : 1

GCA_002293155	DBCQ01000071.1	22	Proteobacteria	TPA_asm: Burkholderiales bacterium UBA954 UBA954_contig_1001, whole genome shotgun sequence
GCA_002359975	DENG01000111.1	21	Proteobacteria	TPA_asm: Polynucleobacter sp. UBA2650 UBA2650_contig_10029, whole genome shotgun sequence
GCA_002342245	DDPQ01000118.1	13	Proteobacteria	TPA_asm: Polynucleobacter sp. UBA2464 UBA2464_contig_1015, whole genome shotgun sequence
	AQUG01000001.1	12	Proteobacteria	Alpha proteobacterium SCGC AAA027-L15 A27L15DRAFT_NODE-unique_1_len_245217.1_C, whole genome shotgun sequence
GCA_000371825	AQUF01000001.1	9	Proteobacteria	Alpha proteobacterium SCGC AAA027-J10 A27J10DRAFT_NODE-unique_1_len_96767.1_C, whole genome shotgun sequence
GCA_002292975	DBCI01000012.1	7	Proteobacteria	TPA_asm: Polynucleobacter sp. UBA962 UBA962_contig_100857, whole genome shotgun sequence
GCA_000364545	AQPD01000001.1	5	Proteobacteria	Alpha proteobacterium SCGC AAA027-C06 YUCDRAFT_NODE-unique_1_len_70157.1_C, whole genome shotgun sequence
GCA_000419465	ATTB01000001.1	3	Proteobacteria	Alpha proteobacterium SCGC AAA028-C07 A28C7DRAFT_NODE-unique_1_len_203146.1_C, whole genome shotgun sequence
GCA_002292945	DBCG01000126.1	2	Proteobacteria	TPA_asm: Proteobacteria bacterium UBA964 UBA964_contig_10071, whole genome shotgun sequence
GCA_000371845	AQUE01000001.1	1	Proteobacteria	Alpha proteobacterium SCGC AAA280-P20 A280P20DRAFT_NODE-unique_1_len_90870.1_C, whole genome shotgun sequence
GCA_002304425	NEUY01000001.1	29	Verrucomicrobiota	Opitutae bacterium Tous-C10FEB Opitutae-Tous-C10FEB-C1, whole genome shotgun sequence
GCA_002304435	NEUZ01000001.1	1	Verrucomicrobiota	Opitutae bacterium Tous-C8FEB Opitutae-Tous-C8FEB-C1, whole genome shotgun sequence

Table S11. Comparison of the *Lyngbya robusta* genome assembly (GCF_000972705) with MegaHit contigs obtained from reads assigned to *Lyngbya robusta* by Kraken2. Only the longest matches are reported. “Lyngbya contig” indicates the assembled MegaHit contigs. Contig “start” and “end”position of match within the contig.

GCF_000972705.2								
Start	End	Lyngbya contig	Lyngbya contig length	Percent identity	Length of match	Strand	Contig start	Contig end
4119641	4142905	k141_980	27750	97.993	23271	rc	1799	25064
1358642	1378938	k141_2289	38169	95.433	20342		17849	38169
6768428	6786921	k141_295	21804	95.834	18580	rc	3001	21536
792193	810338	k141_634	38567	98.529	18147	rc	9806	27949
2169978	2187390	k141_1232	18197	97.986	17425	rc	408	17811
2496809	2513832	k141_315	23145	95.814	17034	rc	105	17126
6791330	6807977	k141_912	25825	97.628	16651	rc	9178	25825
819640	836066	k141_1533	17896	97.675	16428		494	16904
3628652	3644990	k141_651	20331	97.663	16349	rc	3189	19513
1639850	1655676	k141_1730	26891	97.044	15832	rc	5194	20996
5328288	5343867	k141_2121	56377	97.242	15626	rc	7133	22720
7288059	7303475	k141_535	18938	98.425	15425	rc	3047	18434
1288464	1303353	k141_1104	78260	97.422	14896	rc	26701	41589
4494531	4509326	k141_507	25679	97.444	14827	rc	1	14825
3205431	3220117	k141_1665	15224	96.411	14739		1	14695
981482	995486	k141_1323	35836	97.802	14012		14883	28869
5364767	5378652	k141_1564	25626	98.057	13896		9502	23397
3367654	3381249	k141_903	38949	97.662	13599	rc	24821	38417
4750107	4763663	k141_1316	14531	98.326	13557	rc	3	13559
2112310	2125858	k141_2179	20996	98.207	13549		541	14088
7125777	7139311	k141_2091	33929	97.407	13537	rc	20400	33929
1729460	1742842	k141_1307	22670	96.684	13389	rc	851	14216
7192111	7205433	k141_676	22672	97.269	13328	rc	598	13921
205490	218618	k141_1255	23817	95.382	13145		10190	23316
2599589	2612524	k141_251	23718	95.499	12974		564	13525
6215611	6228403	k141_676	22672	97.21	12797	rc	1129	13921
5566682	5579012	k141_27	20312	95.939	12361	rc	7619	19957
648250	660301	k141_1540	24539	97.986	12064		558	12620
2348863	2360673	k141_2113	42131	97.825	11818	rc	28107	39911
327504	339303	k141_136	20903	97.204	11802	rc	8447	20245
5440477	5452208	k141_1668	21470	97.888	11743	rc	4175	15909
4421016	4432703	k141_1220	33116	98.349	11689	rc	8912	20600
375821	387324	k141_253	45713	98.063	11510		8336	19844
5944158	5955625	k141_252	17068	97.821	11473		4629	16100
2741860	2753286	k141_2121	56377	97.149	11434	rc	44944	56345
1857993	1869354	k141_892	33603	94.455	11379		21695	33040
1594163	1605502	k141_1328	20347	97.276	11344	rc	602	11944
5122685	5133875	k141_266	29642	98.186	11192	rc	568	11758
1475989	1487095	k141_2089	15478	97.705	11109	rc	2656	13762
1251182	1262172	k141_753	16050	97.137	11004		139	11119
5104784	5115741	k141_266	29642	97.919	10958	rc	18262	29219
2541511	2552377	k141_351	15874	98.04	10869		5008	15874
1111252	1122059	k141_2328	14315	97.761	10808	rc	599	11373
5874756	5885532	k141_775	25133	96.92	10778		14316	25091
2226652	2237389	k141_555	13802	93.721	10750		1531	12268
2588961	2599546	k141_1769	45432	98.074	10594		34409	45000
5841419	5851863	k141_2131	12749	97.674	10447	rc	2325	12749
1886200	1896598	k141_1229	22942	98.01	10401	rc	9928	20322
5043419	5053766	k141_458	11267	94.275	10375		726	11037
6608292	6618639	k141_2358	10757	97.459	10352	rc	1	10347

Table S12. Summary of results obtained for the MetaBat2 bins using virsorter2 (phage content) and annotation of bins by GTDBtk for the 2017 samples. Only bins with an estimated relative abundance (median MetaBat2 depth value across all contigs within a bin) greater than 5 in the indicated sample (the sample/date used to sort the table) are shown. Values in all samples higher than 5 are shaded grey. Taxonomies identified by GTDBtk are reported at the phylum and genus/species level. The complete list of values including results from checkm are available in Online resource 2 (file vareselake_2.xlsx).

MetaBat2										Virsorter	GTDBtk
bin	Total contiglen	19/7/2017 E	26/7/2017 E	2/8/2017 E	9/8/2017 E	23/8/2017 E	30/8/2017 E	27/9/2017 E	4/10/2017 E		Classification
bin.1	268194	0.2	1.2	29.7	30.9	55.5	24.0	17.4	12.6	phage	
bin.100	3311991	2.3	4.7	1.6	20.9	5.3	7.6	6.9	1.3	phage	Verrucomicrobiota QYQL01 sp007280915
bin.101	6644546	18.4	21.9	8.1	20.7	12.2	30.3	24.3	5.7		Cyanobacteria Lyngbya robusta
bin.102	356020	15.3	18.5	6.8	19.8	10.8	27.5	21.5	4.8		Firmicutes UBA970 sp002292805
bin.104	212566	18.8	10.3	8.6	19.2	9.1	15.7	12.8	4.6		Proteobacteria Fonsibacter ubiquis
bin.103	595305	17.9	8.3	8.4	18.9	7.7	10.9	9.8	4.1		Proteobacteria Polynucleobacter sp002359975
bin.105	925085	11.3	6.2	6.1	17.5	5.9	8.5	7.8	2.8		
bin.106	531269	7.0	5.4	2.5	15.9	6.1	7.5	6.7	2.0		Bacteroidota SYHX01 sp005799025
bin.107	353399	6.7	5.4	2.6	15.6	6.1	7.4	6.8	2.2	phage	
bin.108	233535	6.9	5.3	3.0	14.8	6.0	7.2	6.8	2.3	phage	
bin.109	280254	6.7	5.2	2.9	14.6	5.9	7.2	6.7	2.3	phage	
bin.11	358425	5.9	4.9	2.3	13.1	5.7	6.8	6.5	2.7		
bin.10	246752	2.9	2.1	5.5	4.6	6.3	3.9	3.5	2.0	phage	

MetaBat2										Virsorter	GTDBtk
Bin	Total contiglen	19_7_2017_S	23_8_2017_S	30_8_2017_S	27_9_2017_S	04_10_2017_S	19_7_2017_M	2_8_2017_M	9_8_2017_M		Classification
bin.198	786300	15.7	31.4	32.8	14.4	3.1	0.5	3.2	1.0		Proteobacteria Fonsibacter ubiquis
bin.248	292759	6.1	30.7	29.8	8.6	2.8	0.2	1.4	0.4	phage	
bin.61	362208	4.3	8.9	20.9	4.3	0.7	0.1	1.2	0.3		Actinobacteriota MAG-120802 sp003569145
bin.56	235684	1.7	27.1	20.2	2.7	0.7	0.1	0.5	0.2		
bin.15	213414	0.3	7.0	19.5	6.8	1.0	0.0	0.1	0.0	phage	
bin.155	6696240	7.1	16.4	15.8	7.1	5.3	1.8	11.4	5.5		Cyanobacteria Lyngbya robusta
bin.205	234738	0.2	17.1	15.5	4.4	1.4	0.1	0.1	0.2	phage	
bin.266	2822690	2.6	11.6	12.3	0.5	0.1	0.1	1.5	0.3		Verrucomicrobiota IMCC26134 sp002382525
bin.247	575798	4.0	22.7	11.1	1.2	0.3	0.0	0.9	0.3	phage	
bin.60	838560	17.5	8.9	10.6	7.5	1.3	0.5	4.1	0.7		Actinobacteriota Nanopelagicus sp001437855
bin.255	313612	6.1	13.1	10.1	5.4	1.7	0.1	0.9	0.2	phage	
bin.69	1196598	0.6	4.0	8.4	1.1	0.1	0.0	0.2	0.1		
bin.195	251641	6.5	13.2	7.4	1.3	0.4	0.2	0.9	0.3	phage	
bin.233	228992	0.7	6.8	7.3	1.2	0.3	0.0	0.2	0.2	phage	
bin.88	3283112	1.5	4.3	7.2	2.8	1.5	1.9	2.3	3.9		Verrucomicrobiota QYQL01 sp007280915
bin.127	211018	7.0	7.8	6.7	1.8	1.0	0.3	3.1	0.4	phage	
bin.8	310876	15.2	4.7	6.6	5.6	1.2	1.0	4.9	0.9		Actinobacteriota Planktophila sp001438925
bin.237	1722446	17.3	5.8	6.6	0.3	0.1	0.3	0.9	0.3		Bacteroidota JJ008 sp005791465
bin.147	803712	4.1	3.6	6.6	7.8	0.8	0.6	2.6	0.6		Proteobacteria Methylopumilus universalis
bin.73	702378	8.4	4.4	6.5	9.6	7.8	4.6	8.8	4.4		Actinobacteriota Nanopelagicus sp003569185
bin.92	554580	0.7	1.7	6.1	3.3	0.7	0.3	0.8	0.3		
bin.150	426586	11.5	6.4	6.1	3.5	5.6	4.0	6.6	3.1		Proteobacteria Fonsibacter sp000371825
bin.31	538998	1.3	12.1	5.8	1.3	0.3	0.0	0.2	0.1	phage	
bin.125	352369	10.1	13.7	5.6	1.1	0.3	0.1	2.2	0.4	phage	
bin.39	298041	9.1	7.0	5.6	1.3	0.7	0.2	1.9	0.3	phage	

Table S13. Summary of results obtained for the MetaBat2 bins using virsorter2 (phage content) and annotation of bins by GTDBtk for the 2016 samples. Only bins with an estimated relative abundance (median MetaBat2 depth value across all contigs within a bin) greater than 5 in the indicated sample (the sample/date used to sort the table) are shown. Values in all samples higher than 5 are shaded grey. Taxonomies identified by GTDBtk are reported at the phylum and genus/species level. The complete list of values including results from checkm are available in Online resource 2 (file vareselake_2.xlsx).

MetaBat2							GTDBtk
**Bin**	**Total contiglen**	**31/8/2016 E**	**7/9/2016 E**	**14/9/2016 E**	**21/9/2016 E**	**Virsorter**	**Classification**
bin.28	224055	3.18	13.05	17.86	26.68	phage	
bin.10	845049	4.65	8.4	6.37	14.46		Actinobacteriota Nanopelagicus sp001437855
bin.52	1221917	2.71	5.72	6.14	10.88		Proteobacteria Fonsibacter ubiquis
bin.23	265008	4.58	6.31	4.2	8.97	phage	
bin.36	671716	3.8	6.58	4.47	8.59		Actinobacteriota Nanopelagicus sp003569185
bin.19	543265	5.4	6.28	3.56	8.09	phage	
bin.60	304840	0.58	2.16	7.44	7.38	phage	
bin.12	1042573	2.17	7.05	3.97	7.29	phage	
bin.31	3920867	1.44	3.7	5.08	6.37	phage	
bin.63	4515768	1.57	4.94	3.86	6.17	phage	
bin.71	238065	2.55	4.53	2.41	5.89		
bin.46	297689	1.91	3.32	2.75	5.35		
bin.72	1596733	2.81	3.51	1.87	5.35		Proteobacteria Polynucleobacter sp002359975

MetaBat2							GTDBtk
**Bin**	**Total contiglen**	**7/9/2016 S**	**14/9/2016 S**	**21/9/2016 S**	**31/8/2016 M**	**Virsorter**	**Classification**
bin.49	2720869	2.74	56.56	52.65	1.97		Cyanobacteria Synechococcus_D
bin.48	3431136	0.53	24.6	0.02	0.01		Bdellovibrionota Silvanigrellaceae
bin.3	386814	0.15	13.82	0.68	0.31		
bin.43	322609	0.2	10.37	0.7	4.39	phage	
bin.5	318630	3.44	7.15	6.11	0.05	phage	
bin.47	396409	8.82	7.11	22.86	12.33		
bin.57	204091	1.79	5.44	7.3	0.22		
bin.58	4476435	6.24	5.23	4.17	0.26		Cyanobacteri Snowella

Table S14. Conservation of nitrogenase and nitrogen-fixing genes from *Lyngbya robusta* in the MetaBat2 bin assigned to *Lyngbya robusta*. Percent sequence identity and match length values shown were obtained with DIAMOND (blastx option) comparing the contigs in the MetaBat2 bin against the *Lyngbya robusta* proteome as defined in NZ_LATL02000057.1.

NZ_LATL02000154.1 Lyngbya robusta genome							MetaBat2 bin assigned to Lyngbya robusta	
Start pos	End pos	strand	NCBI Accession	Gene	Length (aa)	Description	% sequence identity	Match length (aa)
3858	4058	-	WP_046280725.1	nifT	67	putative nitrogen fixation protein NifT	97	66
4220	4486	-	WP_046280720.1		89	nitrogen fixation protein NifZ	98.9	88
4473	5606	-	WP_046280726.1	nifV	378	homocitrate synthase	99.7	377
5888	6100	-	WP_046280727.1		71	DUF2949 domain-containing protein	100	70
6850	7146	-	WP_046280721.1		99	hypothetical protein	100	98
7208	7939	-	WP_046280722.1	cysE	244	serine O-acetyltransferase	100	174
9259	10719	+	WP_046280723.1	nifB	487	nitrogenase cofactor biosynthesis protein NifB	99.4	487
11042	12244	+	WP_046280728.1	nifS	401	cysteine desulfurase NifS	99.3	400
12454	13350	+	WP_046276697.1	nifU	299	Fe-S cluster assembly protein NifU	97.7	298
13959	14861	+	WP_046276698.1	nifH	301	nitrogenase iron protein	98	300
14986	16434	+	WP_046276713.1	nifD	483	nitrogenase molybdenum-iron protein alpha chain	96.7	482
16659	18197	+	WP_200906277.1	nifK	513	nitrogenase molybdenum-iron protein subunit beta	96.9	511
18365	18691	+	WP_046276700.1		109	Mo-dependent nitrogenase C-terminal domain-containing protein	100	108
18740	20161	+	WP_046276701.1	nifE	474	nitrogenase iron-molybdenum cofactor biosynthesis protein NifE	98.7	473
20263	21618	+	WP_046276702.1	nifN	452	nitrogenase iron-molybdenum cofactor biosynthesis protein NifN	99.6	451
21779	22264	+	WP_046276703.1		162	DUF269 domain-containing protein	96.3	161
22277	22687	+	WP_049559465.1	nifX	137	nitrogen fixation protein NifX	94.2	137
22820	23299	+	WP_082172436.1		160	NifX-associated nitrogen fixation protein	100	159
23374	23607	+	WP_046276705.1		78	hypothetical protein	100	77
23604	23942	+	WP_046276706.1	nifW	113	nitrogenase-stabilizing/protective protein NifW	93.8	112

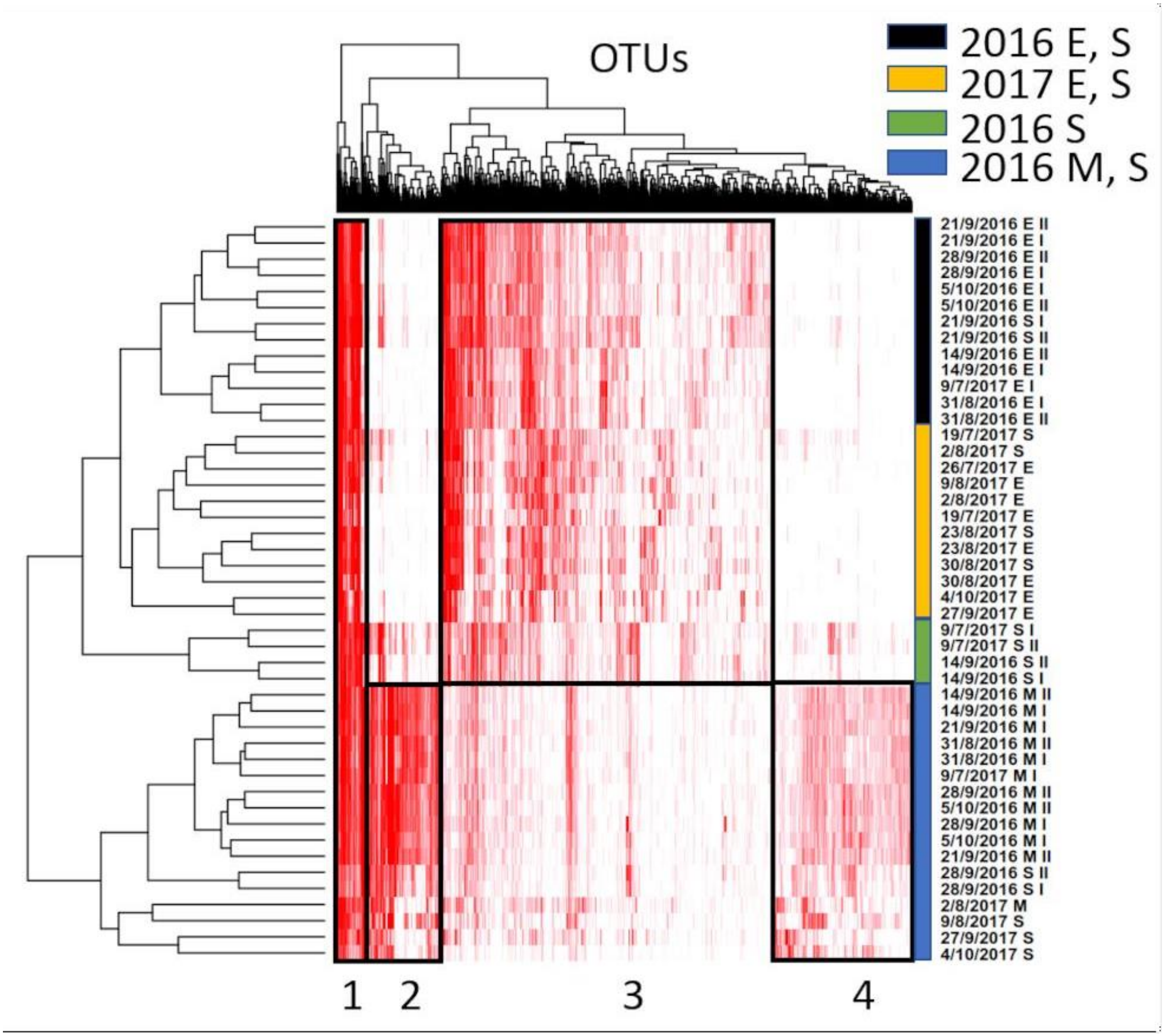

Fig. S1. Operational taxonomy units (OTUs) clustering of 16S samples. Only OTUs with total abundance higher than 100 across all samples were included. Values shown are log2 of OTUabundance (OTU abundances of zero were set 1). E = Epi, S = 2.5x Secchi, M = Meso. Four large clusters are indicated by numbers on the bottom. I and II are duplicates of the same sample

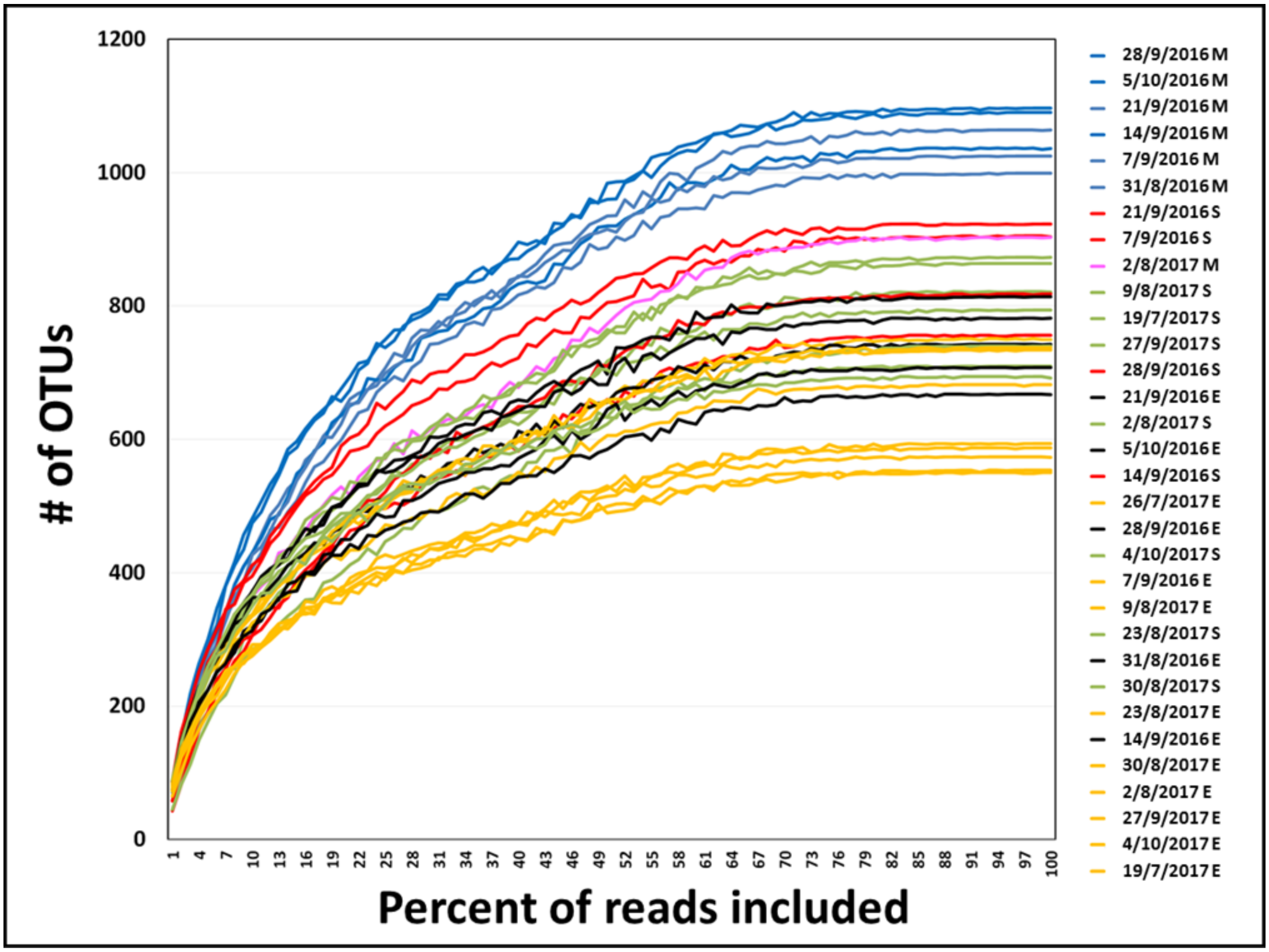

Fig. S2. Rarefaction curve analysis of operational taxonomy units (OTUs). Samples names shown on the right are ordered from top to bottom according to the maximum number of OTUs present. Colour code is yellow (2017 Epi - E), black (2016 E), red (2016 2.5x Secchi - S), green (2017 S), magenta (2017 Meso - M), blue (2016 M). 100% on the horizontal axis corresponds to 22,000 rarefied amplicon sequences

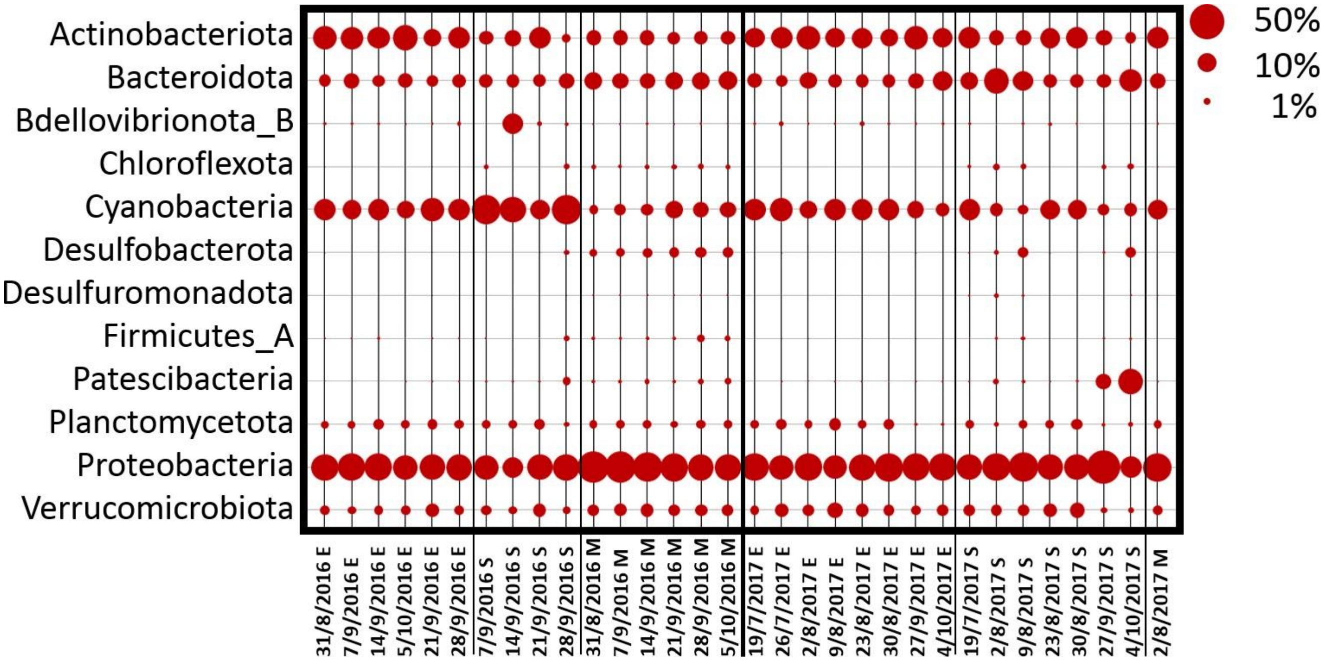

Fig. S3. Composition of the microbial community at the phylum level as determined from 16S data. The community composition is shown for all the water samples collected during the sampling period in Lake Varese at the three different depths (Epi - E - 0.5 m; 2.5x Secchi - S - and Meso - M - 13 m). Samples were sequenced for 16S rRNA (V3-V4 region) and analysed at the phylum level. Secchi depths were measured at each site using a Secchi disk and values were multiplied by 2.5. The M depth corresponded to the S depth on 31/8/2016 and 5/10/2016. Results are expressed as percent of all 16S amplicons. For 2016 replicate samples, the average value is shown. Only phyla observed with higher than 1% abundance are shown. Circles shown represent percent values within the fraction of taxonomy assigned shotgun sequences (circles shown at the top right of the figure represent example percent values)

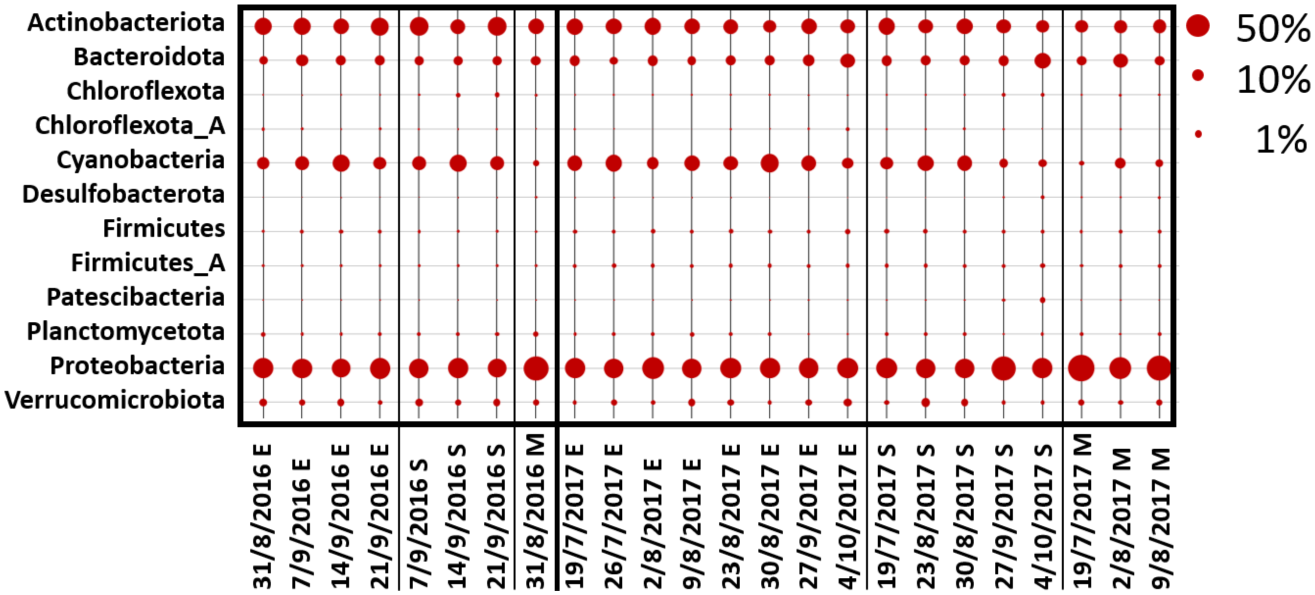

Fig. S4. Community composition at the phylum level as determined from the shotgun data. The community composition is shown for all the water samples collected during the sampling period in Lake Varese at the three different depths (Epi - E - 0.5 m; 2.5x Secchi - S - and Meso - M - 13 m). Samples were analysed for shotgun sequencing. The figure shows variations in the microbial community at the phylum level. Secchi depths were measured at each site using a Secchi disk and values multiplied by 2.5. The M depth corresponded to the S depth on 31/8/2016. Circles shown represent percent values within the fraction of taxonomy assigned shotgun sequences (circles shown at the top right of the figure represent example percent values)

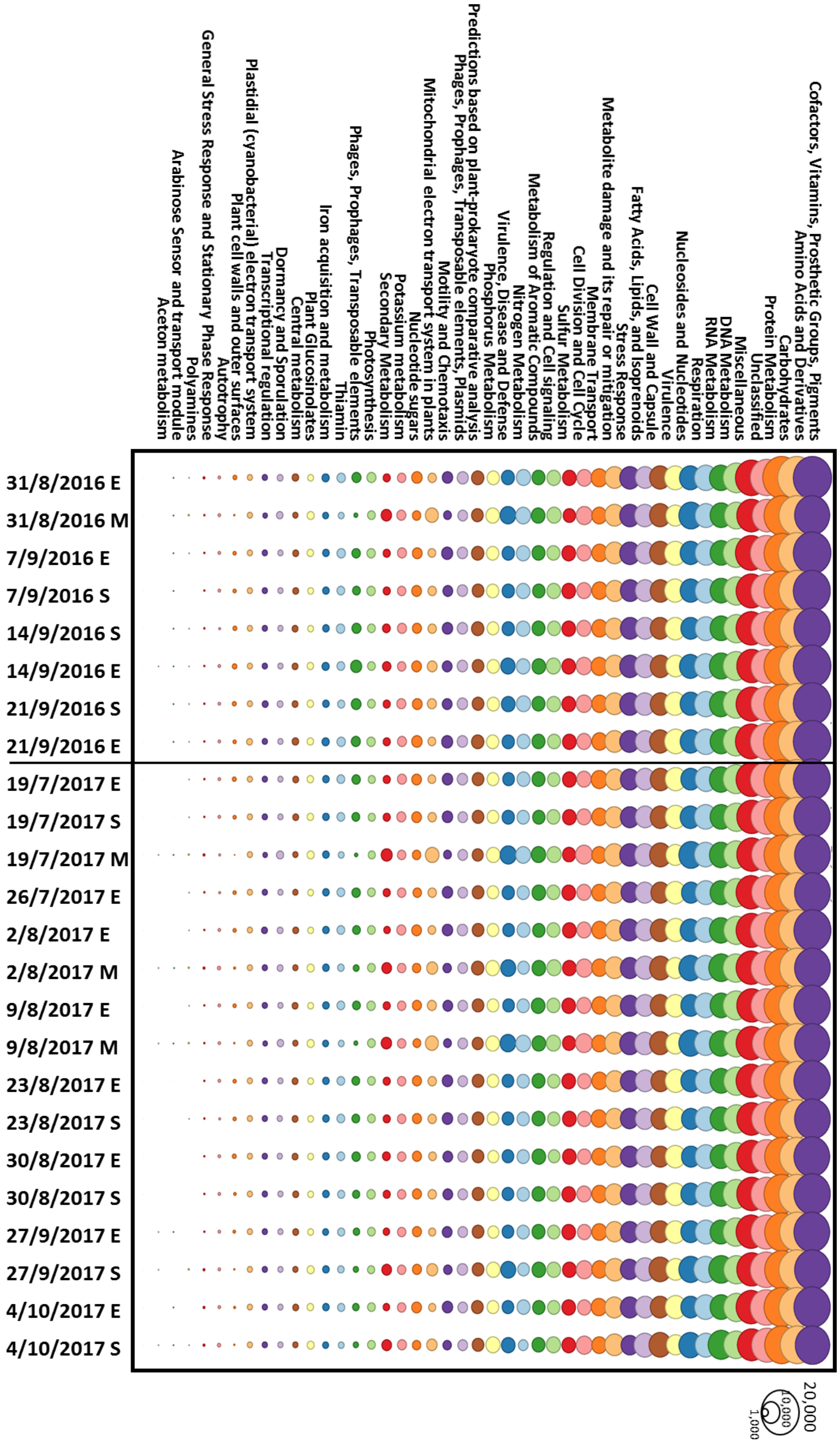

Fig. S5. Functional profile (SEED level 1) of the microbial community (shotgun samples). A representation of the SEED level 1 functional profiles as determined by MEGAN6 from DIAMOND blastx searches. Circles shown are proportional to the number of matches against the indicated SEED level 1 class. E stands for Epi, M for Meso and S for 2.5x Secchi

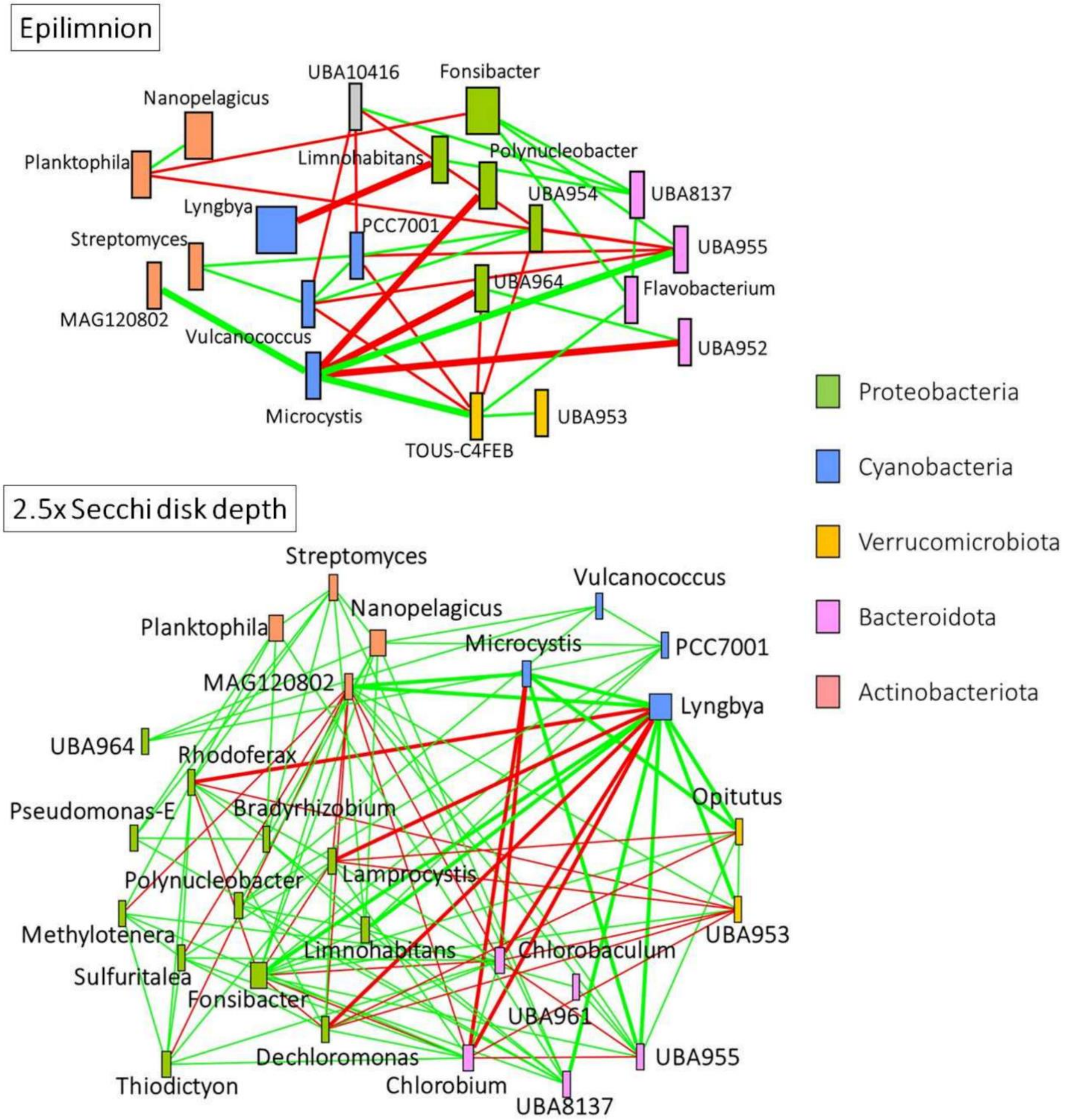

Fig. S6. Epilimnion and 2.5x Secchi depth co-occurence network as calculated by Conet and Cytoscape based on the 2017 genus level Kraken2 results (genera with relative abundance >= 1%). Connections involving *Lyngbya* or *Microcystis* are indicated by thicker lines. Green lines indicate positive interactions (co-occurrence) while red lines indicate negative interactions (mutual exclusion). Genera belonging to the same phylum are indicated by the color code on the right
